# Supplementary material for: Platinum(IV) Prodrugs Incorporating an Indole-Based Derivative, 5-Benzyloxyindole-3-Acetic Acid in the Axial Position Exhibit Prominent Anticancer Activity
Source: Int J Mol Sci. 2024 Feb 11;25(4):2181. doi: 10.3390/ijms25042181 (PMC10888562; doi:10.3390/ijms25042181)
Supplement: Supplementary file 1 [file ijms-25-02181-s001.zip › ijms-2773799-supplementary.pdf]

SUPPLEMENTARY MATERIALS

Platinum(IV) Prodrugs Incorporating an Indole-Based Derivative, 5-Benzyloxyindole-3-Acetic Acid in the Axial Position Exhibit Prominent Anticancer Activity

Angelico D. Aputen,[1] Maria George Elias,[1,2] Jayne Gilbert,[3] Jennette A. Sakoff,[3] Christopher P. Gordon,[1] Kieran F. Scott[2,4] and Janice R. Aldrich-Wright [1*]

[1] School of Science, Western Sydney University, Sydney, NSW 2751, Australia

[2] Ingham Institute, Sydney, NSW 2170, Australia

[3] Calvary Mater Newcastle Hospital, Newcastle, NSW 2298, Australia

[4] School of Medicine, Western Sydney University, Sydney, NSW 2751, Australia

[*] Correspondence: j.aldrich-wright@westernsydney.edu.au

Table of Contents

**Figure S1**	HPLC chromatogram of NHS-5B3A at 254 nm with product peak trace at 11.8 min.	5
**Figure S2**	^{1}H-NMR spectrum of NHS-5B3A in DMSO-d_6.	5
**Figure S3**	2D-COSY spectrum of NHS-5B3A in DMSO-d_6, highlighting the coupling of indole protons.	6
**Figure S4**	^{1}H-NMR spectrum of 5B3A in DMSO-d_6.	6
**Figure S5**	2D-COSY spectrum of NHS-5B3A in DMSO-d_6.	7
**Figure S6**	HPLC chromatogram of **P-5B3A** at 254 nm.	7
**Figure S7**	HPLC chromatogram of **5-5B3A** at 254 nm.	8
**Figure S8**	HPLC chromatogram of **56-5B3A** at 254 nm.	8
**Figure S9**	^{1}H-NMR spectrum of **P-5B3A** in DMSO-d_6.	9
**Figure S10**	^{1}H-NMR spectrum of **5-5B3A** in DMSO-d_6.	10
**Figure S11**	^{1}H-NMR spectrum of **56-5B3A** in DMSO-d_6.	11
**Figure S12**	2D-COSY spectrum of **P-5B3A** in DMSO-d_6.	12
**Figure S13**	2D-COSY spectrum of **5-5B3A** in DMSO-d_6.	13
**Figure S14**	2D-COSY spectrum of **56-5B3A** in DMSO-d_6.	14
**Figure S15**	^{1}H-^{195}Pt-HMQC spectrum of **P-5B3A** in DMSO-d_6 at 400 ppm.	15
**Figure S16**	^{1}H-^{195}Pt-HMQC spectrum of **P-5B3A** in DMSO-d_6 at –2800 ppm.	16
**Figure S17**	^{1}H-^{195}Pt-HMQC spectrum of **5-5B3A** in DMSO-d_6 at 400 ppm.	17
**Figure S18**	^{1}H-^{195}Pt-HMQC spectrum of **5-5B3A** in DMSO-d_6 at –2800 ppm.	18
**Figure S19**	^{1}H-^{195}Pt-HMQC spectrum of **56-5B3A** in DMSO-d_6 at 400 ppm.	19
**Figure S20**	^{1}H-^{195}Pt-HMQC spectrum of **56-5B3A** in DMSO-d_6 at –2800 ppm.	20
**Figure S21**	UV spectra of **P-5B3A** and plot curves at 204, 279 and 304 nm.	21
**Figure S22**	UV spectra of **5-5B3A** and plot curves at 204, 286 and 310 nm.	22
**Figure S23**	UV spectra of **56-5B3A** and plot curves at 206, 250, 291 and 315 nm.	23
**Figure S24**	CD spectrum of **P-5B3A** (0.05 mM) acquired after 30 accumulations.	24
**Figure S25**	CD spectrum of **5-5B3A** (0.05 mM) acquired after 30 accumulations.	24
**Figure S26**	CD spectrum of **56-5B3A** (0.05 mM) acquired after 30 accumulations.	25
**Figure S27**	Plots of log k versus concentration of the organic solvent, CH_3CN to determine the chromatographic lipophilicity index, log k_w of **P-5B3A**, **5-5B3A** and **56-5B3A**.	26

**Figure S28**	Preliminary 1D-^{195}Pt-NMR spectra of **P-5B3A** with PBS in DMSO-d_6 within the regions of 400 and –2800 ppm and at 37 °C	27
**Figure S29**	Preliminary 1D-^{195}Pt-NMR spectra of **5-5B3A** with PBS in DMSO-d_6 within the regions of 400 and –2800 ppm and at 37 °C.	28
**Figure S30**	Preliminary 1D-^{195}Pt-NMR spectra of **56-5B3A** with PBS in DMSO-d_6 within the regions of 400 and –2800 ppm and at 37 °C.	29
**Figure S31**	^{1}H-NMR spectra of **P-5B3A** with PBS and AsA in DMSO-d_6 at 37 °C, in different time intervals, displaying the shift in chemical multiplicity. **T** is time in min.	30
**Figure S32**	1D-^{195}Pt-NMR spectra of **P-5B3A** with PBS and AsA in DMSO-d_6, within the regions of 400 and –2800 ppm at 37 °C, displaying its complete reduction after 2 h from the final ^{1}H-NMR experiment.	31
**Figure S33**	^{1}H-NMR spectra of **5-5B3A** with PBS and AsA in DMSO-d_6 at 37 °C, in different time intervals, displaying the shift in chemical multiplicity. **T** is time in min.	32
**Figure S34**	1D-^{195}Pt-NMR spectra of **5-5B3A** with PBS and AsA in DMSO-d_6, within the regions of 400 and –2800 ppm at 37 °C, displaying its complete reduction after 2 h from the final ^{1}H-NMR experiment.	33
**Figure S35**	^{1}H-NMR spectra of **56-5B3A** with PBS and AsA in DMSO-d_6 at 37 °C, in different time intervals, displaying the shift in chemical multiplicity. **T** is time in min.	34
**Figure S36**	1D-^{195}Pt-NMR spectra of **56-5B3A** with PBS and AsA in DMSO-d_6, within the regions of 400 and –2800 ppm at 37 °C, displaying its complete reduction after 1 h from the final ^{1}H-NMR experiment.	35
**Figure S37**	Combined HPLC chromatograms acquired for **P-5B3A**, **5-5B3A** and **56-5B3A** at 254 nm after treatment with PBS at 37 °C, at 0, 36 and 72 h.	36
**Figure S38**	Combined HPLC chromatograms acquired for **P-5B3A**, **5-5B3A** and **56-5B3A** at 254 nm after treatment with PBS at 37 °C, at 120 h and 1 week.	37
**Figure S39**	ROS production upon treatment with **P-5B3A**, **5-5B3A** and **56-5B3A**, and cisplatin in HT29 colon cells at 0, 0.25, 0.5, 1, 3, 6, 12, 24, 48 and 72 h. TBHP: tert-butyl hydroperoxide. Data points denote mean ± SEM. *n* = 3 from three independent experiments where samples were run in triplicates.	38
**Figure S40**	ROS generation in HT29 colon cells after treatment with **P-5B3A**, **5-5B3A** and **56-5B3A**, 5B3A, the equivalent platinum(II) precursors (**PHEN*SS***, **5ME*SS*** and **56ME*SS***) and platinum(IV)	39

	scaffolds (**PHEN*SS*(IV)(OH)**$_2$, **5ME*SS*(IV)(OH)**$_2$ and **56ME*SS*(IV)(OH)**$_2$), and cisplatin at 24, 48 and 72 h. Data are expressed in RFU values. The RFU values of the platinum(II) precursors (**PHEN*SS***, **5ME*SS*** and **56ME*SS***) and the platinum(IV) scaffolds (**PHEN*SS*(IV)(OH)**$_2$, **5ME*SS*(IV)(OH)**$_2$ and **56ME*SS*(IV)(OH)**$_2$) were included in the diagrams for comparison. Data points signify mean ± SEM. $n = 3$ from three independent tests where samples were achieved in triplicate. Significance was identified by Tukey's multiple comparisons test for multiple comparisons compared with cisplatin, as measured by one-way ANOVA.	
**Figure S41**	The detected MtMP changes in HT29 colon cells after treatment with **P-5B3A**, **5-5B3A** and **56-5B3A**, 5B3A, the equivalent platinum(II) precursors (**PHEN*SS***, **5ME*SS*** and **56ME*SS***) and platinum(IV) scaffolds (**PHEN*SS*(IV)(OH)**$_2$, **5ME*SS*(IV)(OH)**$_2$ and **56ME*SS*(IV)(OH)**$_2$), and cisplatin at 24, 48 and 72 h. Data are expressed in RFU values. Data points signify mean ± SEM. $n = 3$ from three independent tests where samples were achieved in triplicate. Significance was identified by Tukey's multiple comparisons test for multiple comparisons. Group differences were considered statistically significant if * $p < 0.05$, ** $p < 0.01$, *** $p < 0.001$ and **** $p < 0.0001$ compared with cisplatin, as measured by one-way ANOVA.	40
**Figure S42**	HDAC activity upon treatment with **P-5B3A**, **5-5B3A** and **56-5B3A**, 5B3A, the equivalent platinum(II) precursors (**PHEN*SS***, **5ME*SS*** and **56ME*SS***) and platinum(IV) scaffolds (**PHEN*SS*(IV)(OH)**$_2$, **5ME*SS*(IV)(OH)**$_2$ and **56ME*SS*(IV)(OH)**$_2$), and cisplatin at 72 h. Data points signify mean ± SEM. n = 3 from three independent experiments where samples were conducted in triplicate. Significance was identified by Tukey's multiple comparisons test for multiple comparisons. Group differences were considered statistically significant if ** $p < 0.01$ and *** $p < 0.001$ compared with cisplatin, as measured by one-way ANOVA.	41

HPLC Chromatogram of NHS-5B3A

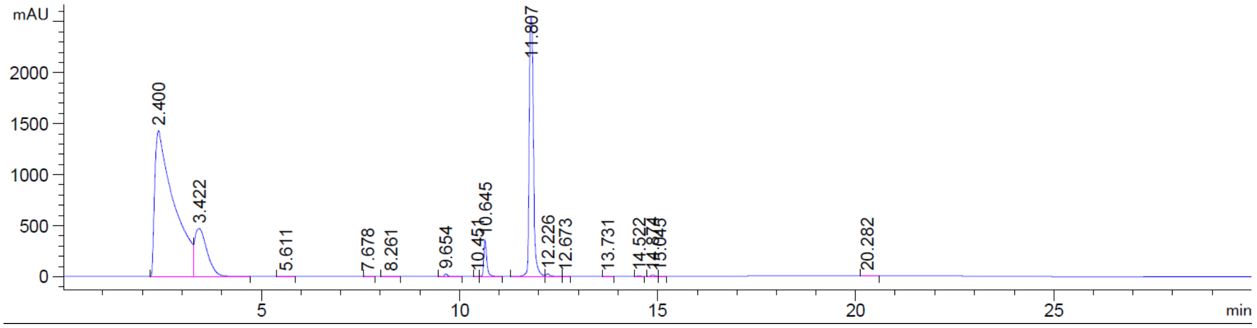

Figure S1: HPLC chromatogram of NHS-5B3A at 254 nm with product peak trace at 11.8 min.

^{1}H-NMR Spectra of NHS-5B3A

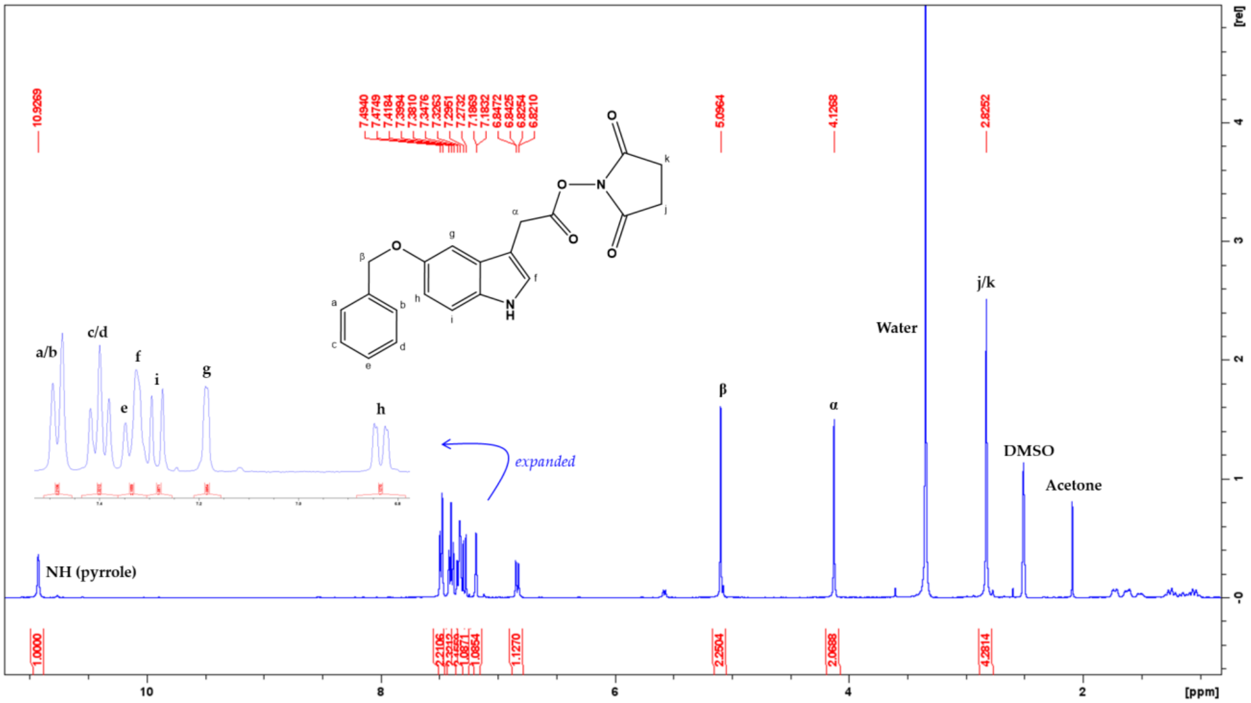

Figure S2: ^{1}H-NMR spectrum of NHS-5B3A in DMSO-d_6.

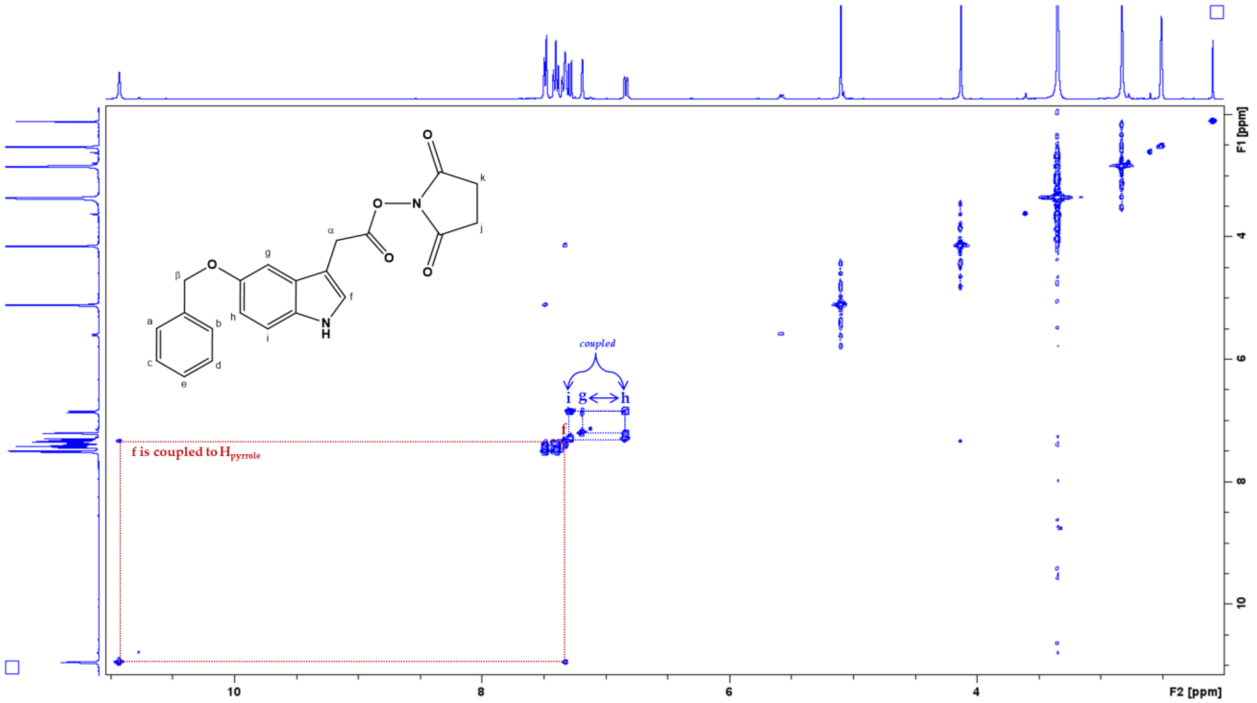

Figure S3: 2D-COSY spectrum of NHS-5B3A in DMSO-d₆, highlighting the coupling of indole protons.

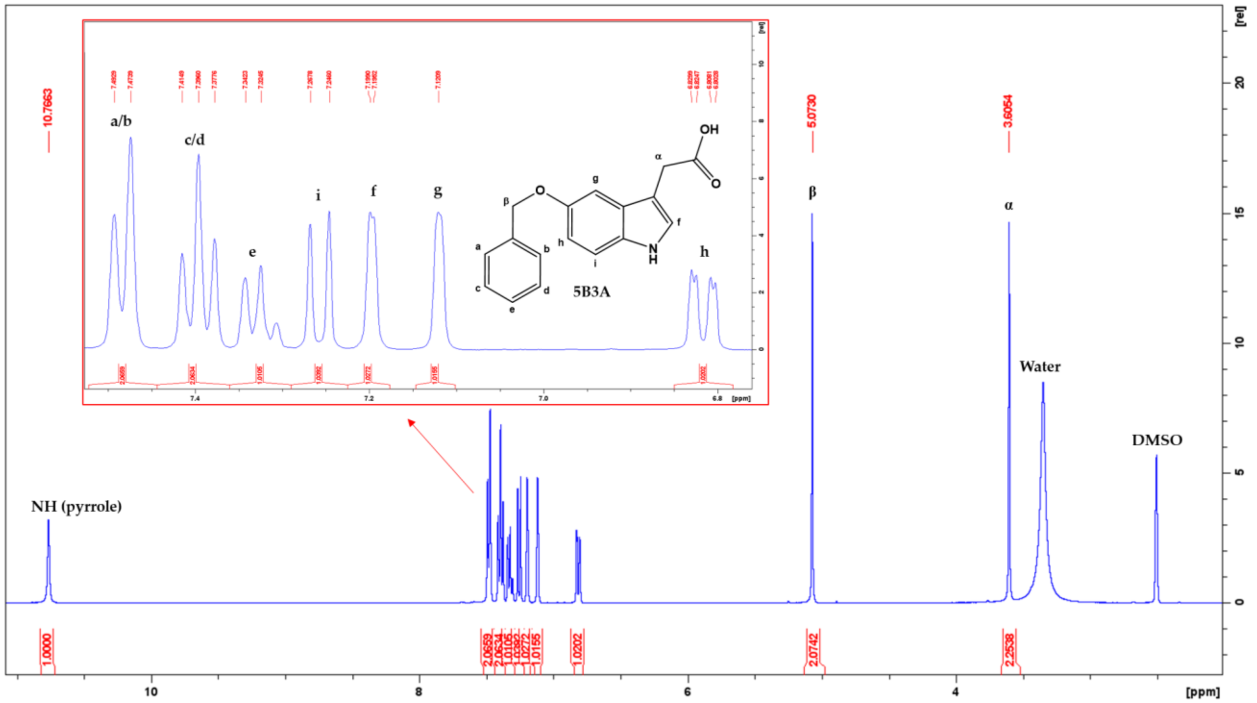

Figure S4: ^{1}H-NMR spectrum of 5B3A in DMSO-d₆.

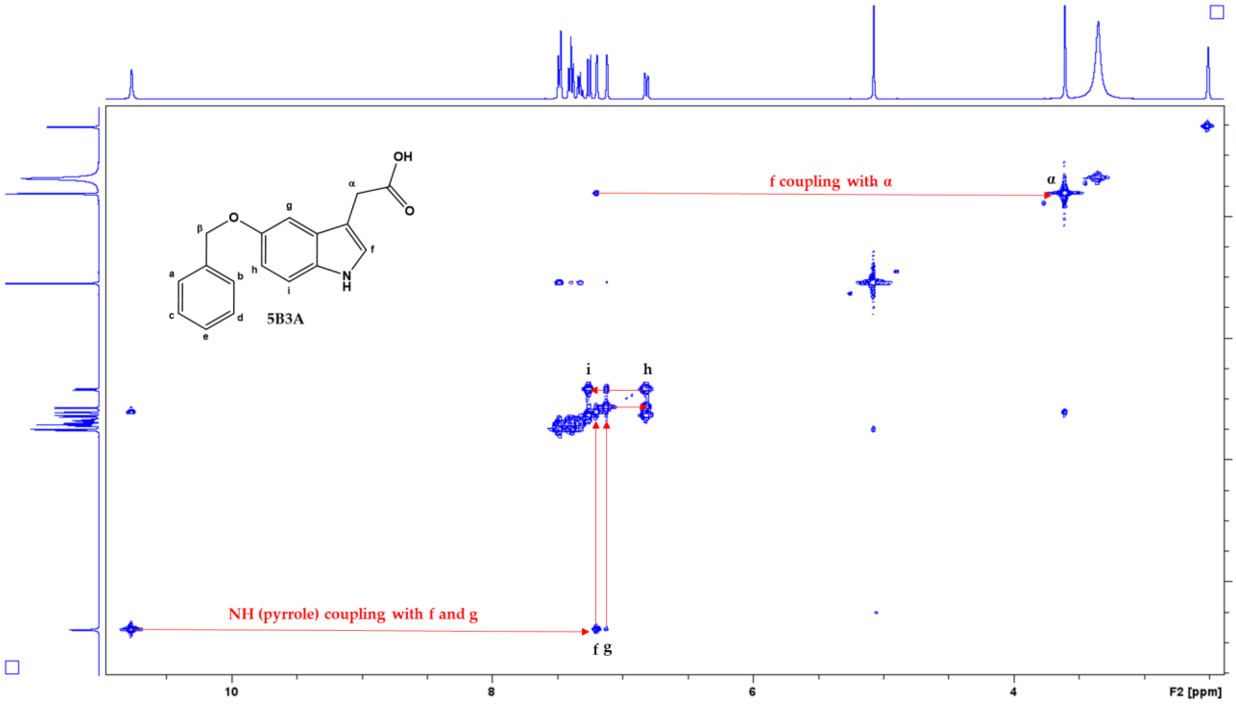

Figure S5: 2D-COSY spectrum of 5B3A in DMSO-d_6, highlighting the coupling of indole protons.

HPLC Chromatograms of P-5B3A, 5-5B3A and 56-5B3A

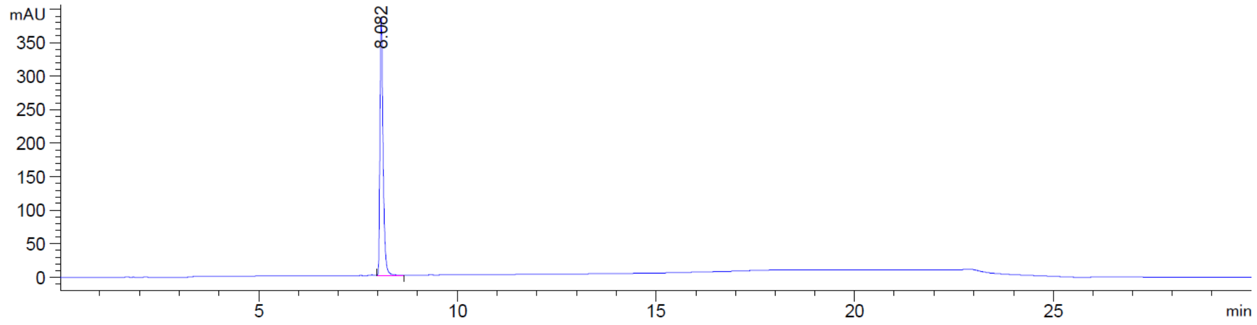

Figure S6: HPLC chromatogram of **P-5B3A** at 254 nm.

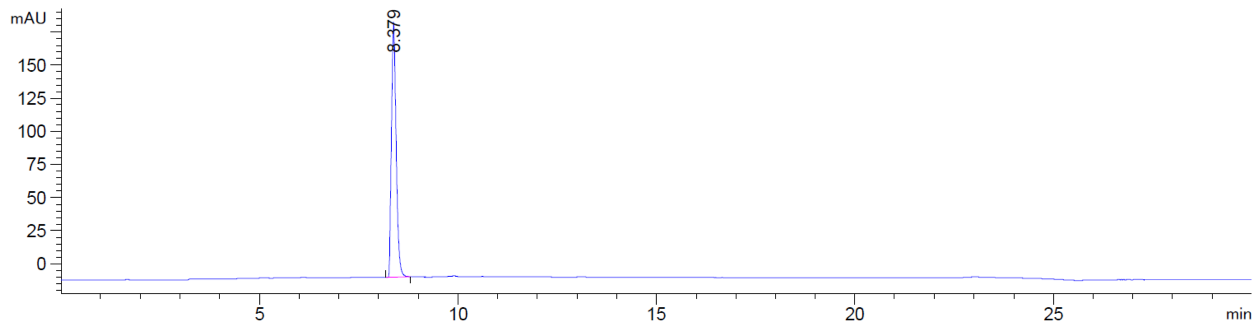

Figure S7: HPLC chromatogram of **5-5B3A** at 254 nm.

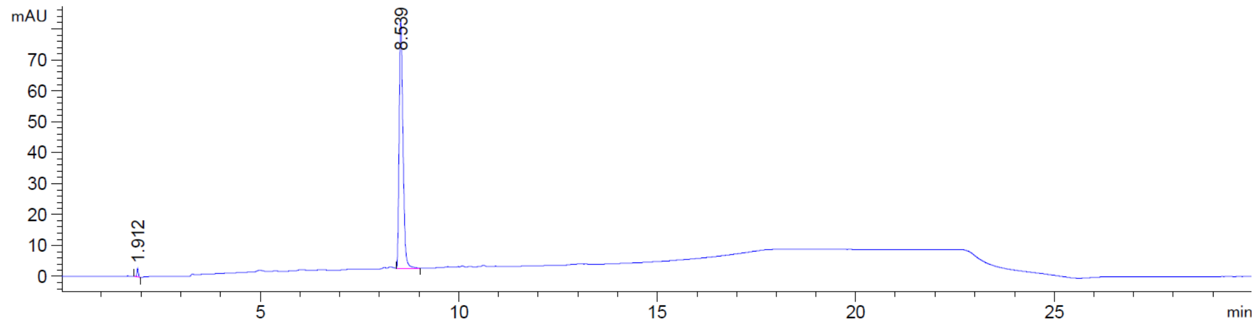

Figure S8: HPLC chromatogram of **56-5B3A** at 254 nm.

^{1}H-NMR Spectra of P-5B3A, 5-5B3A and 56-5B3A

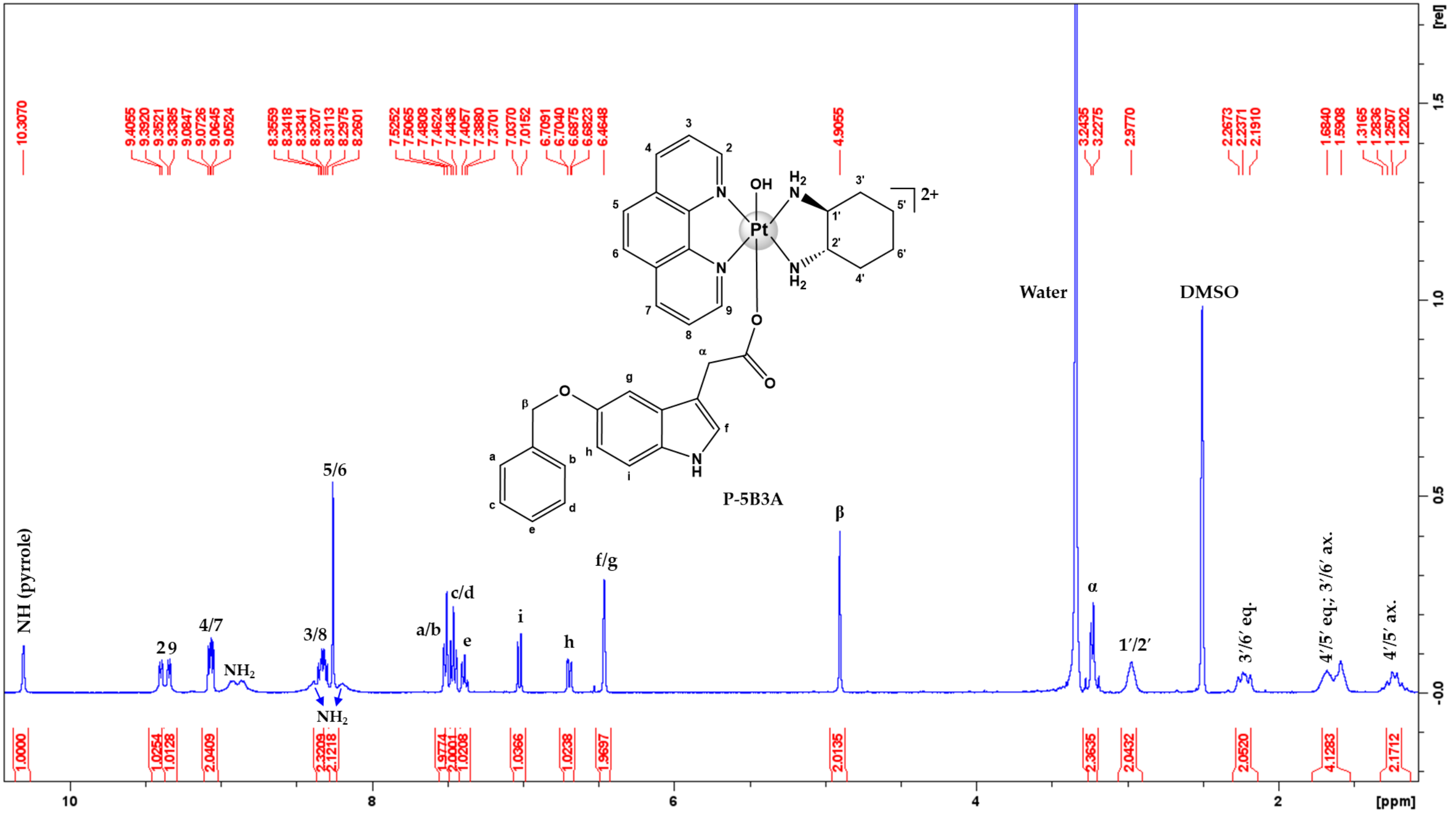

Figure S9: ^{1}H-NMR spectrum of **P-5B3A** in DMSO-d_6.

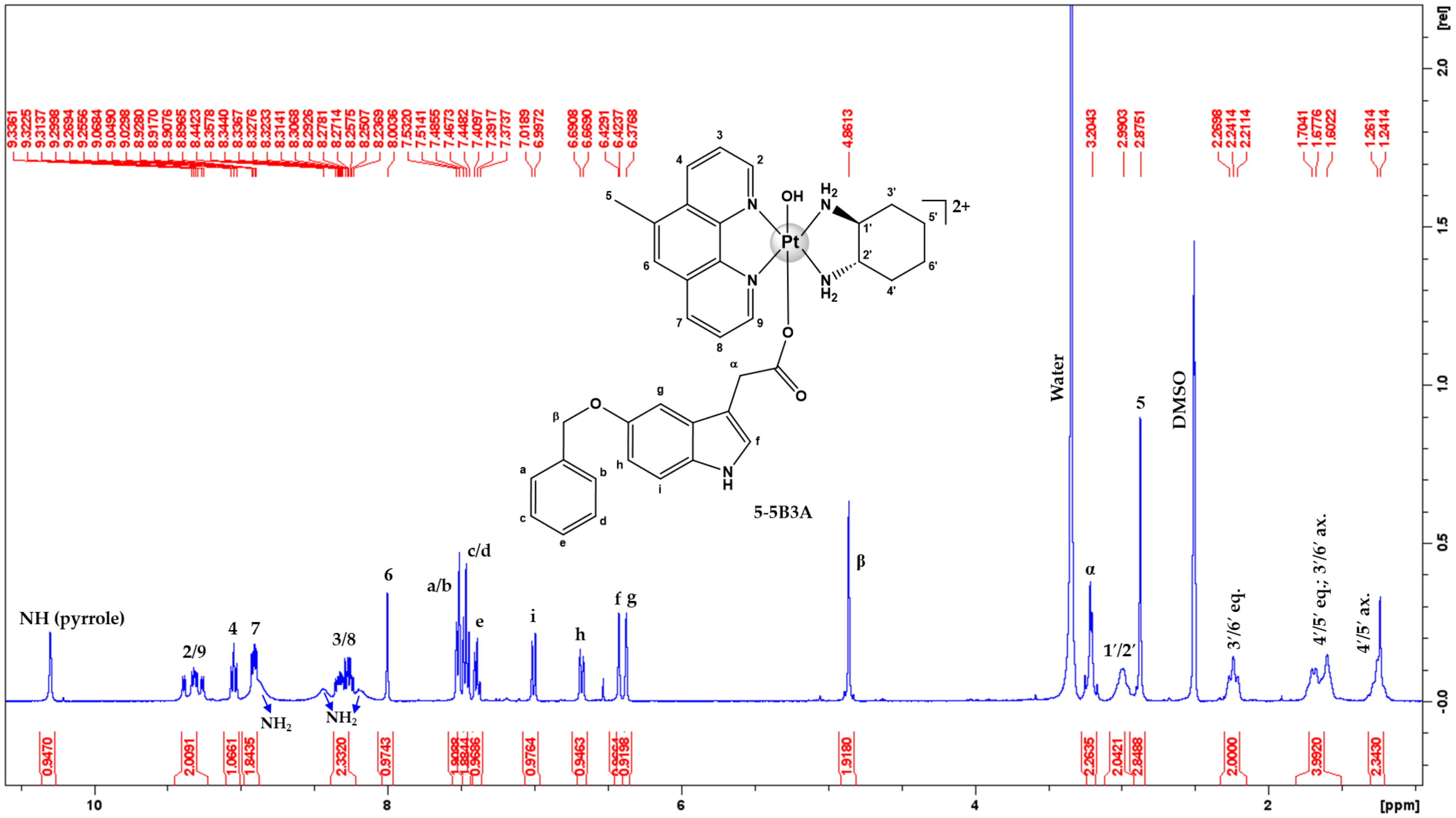

Figure S10: ^{1}H-NMR spectrum of **5-5B3A** in DMSO-d_6.

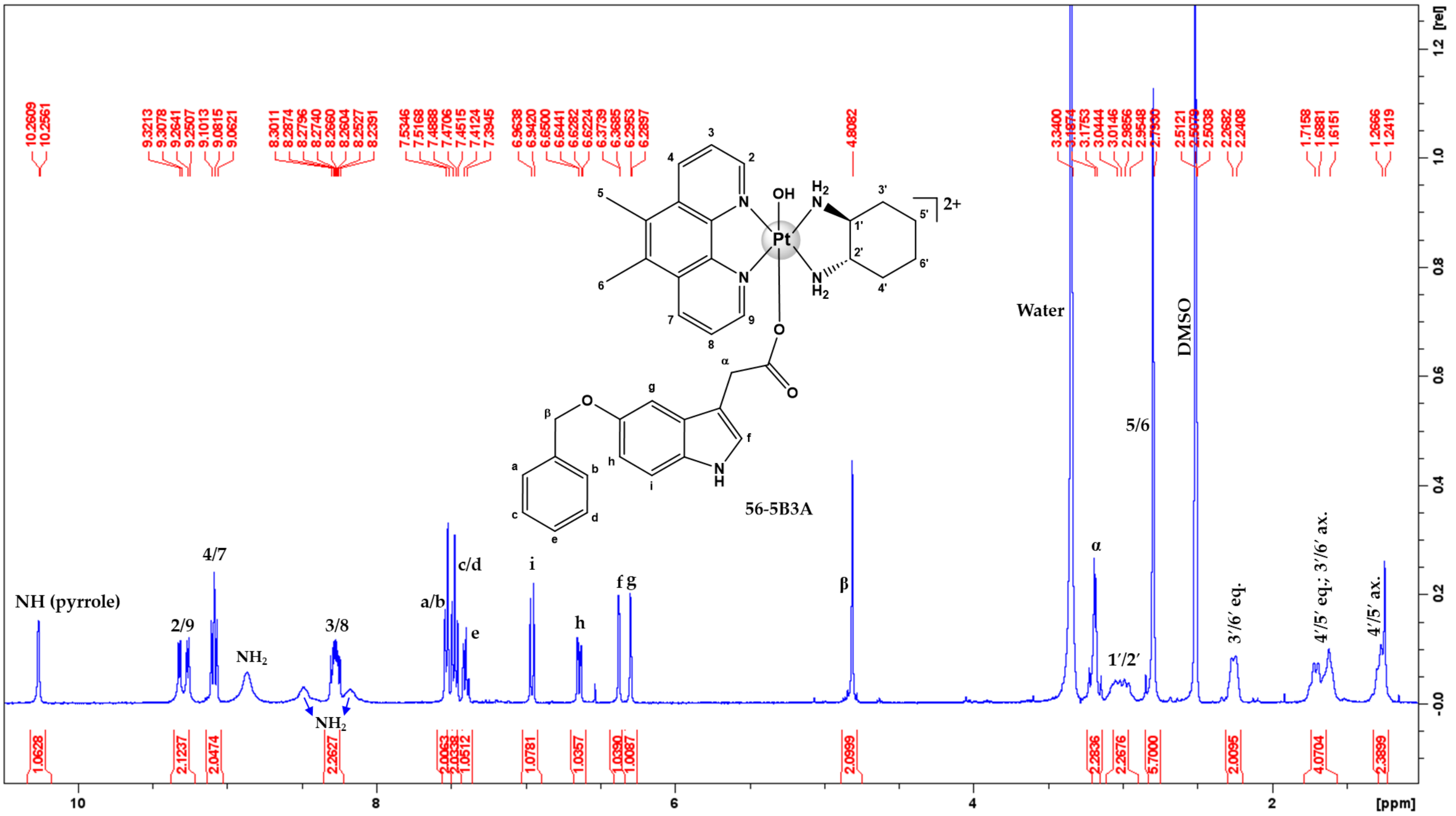

Figure S11: ^{1}H-NMR spectrum of **56-5B3A** in DMSO-d_6.

2D-COSY Spectra of P-5B3A, 5-5B3A and 56-5B3A

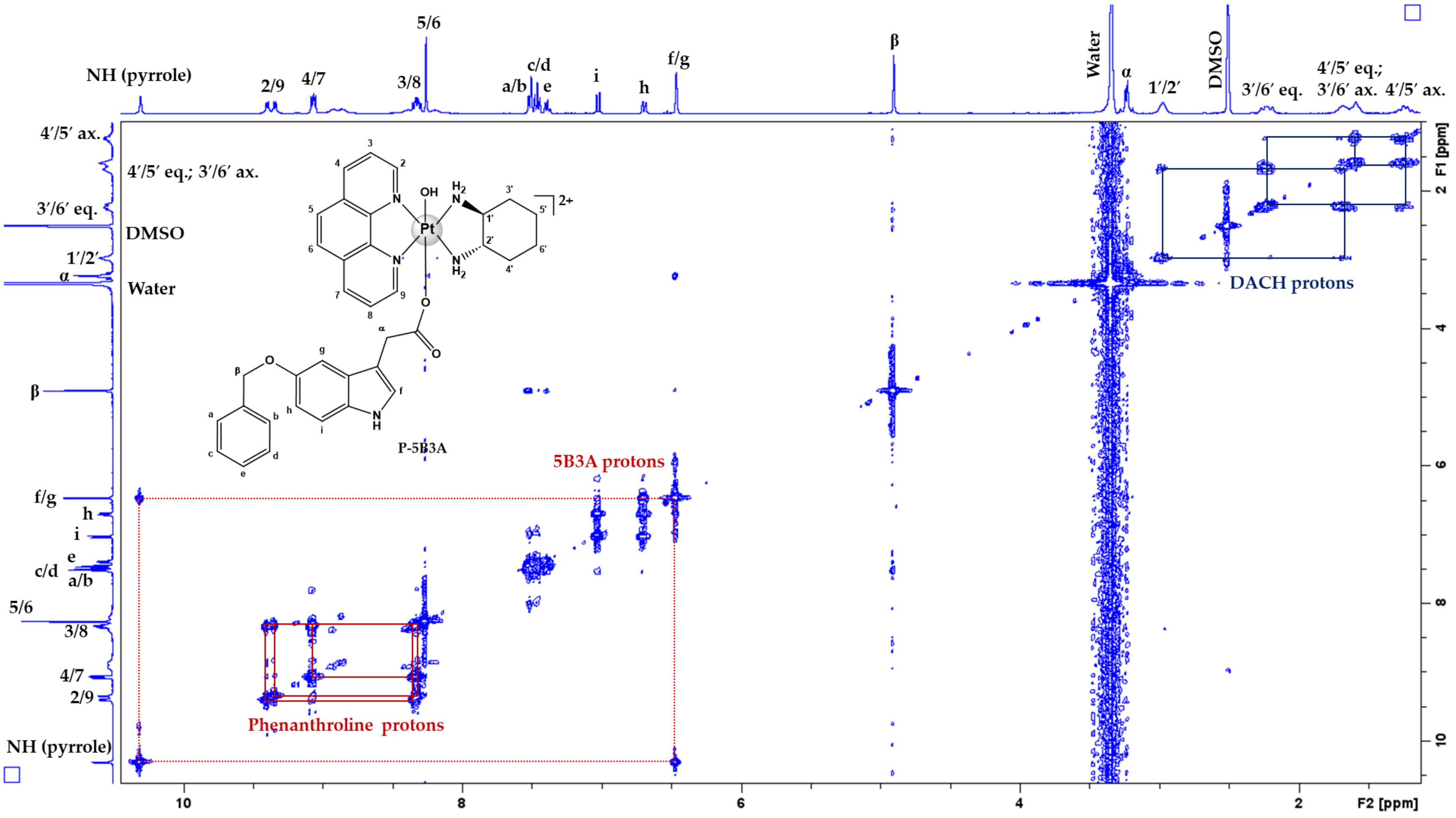

Figure S12: 2D-COSY spectrum of **P-5B3A** in DMSO-d6.

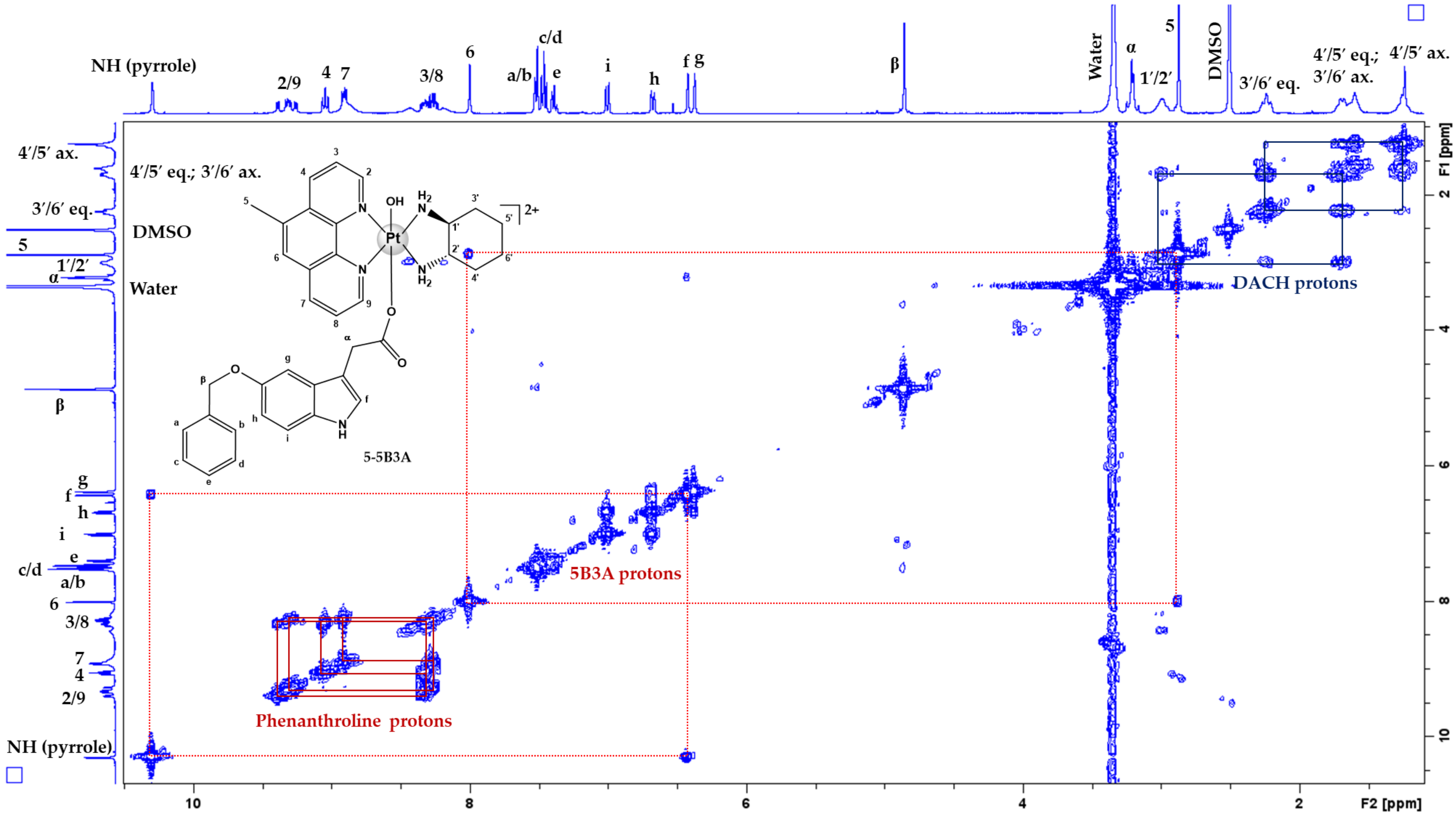

Figure S13: 2D-COSY spectrum of of **5-5B3A** in DMSO-d6.

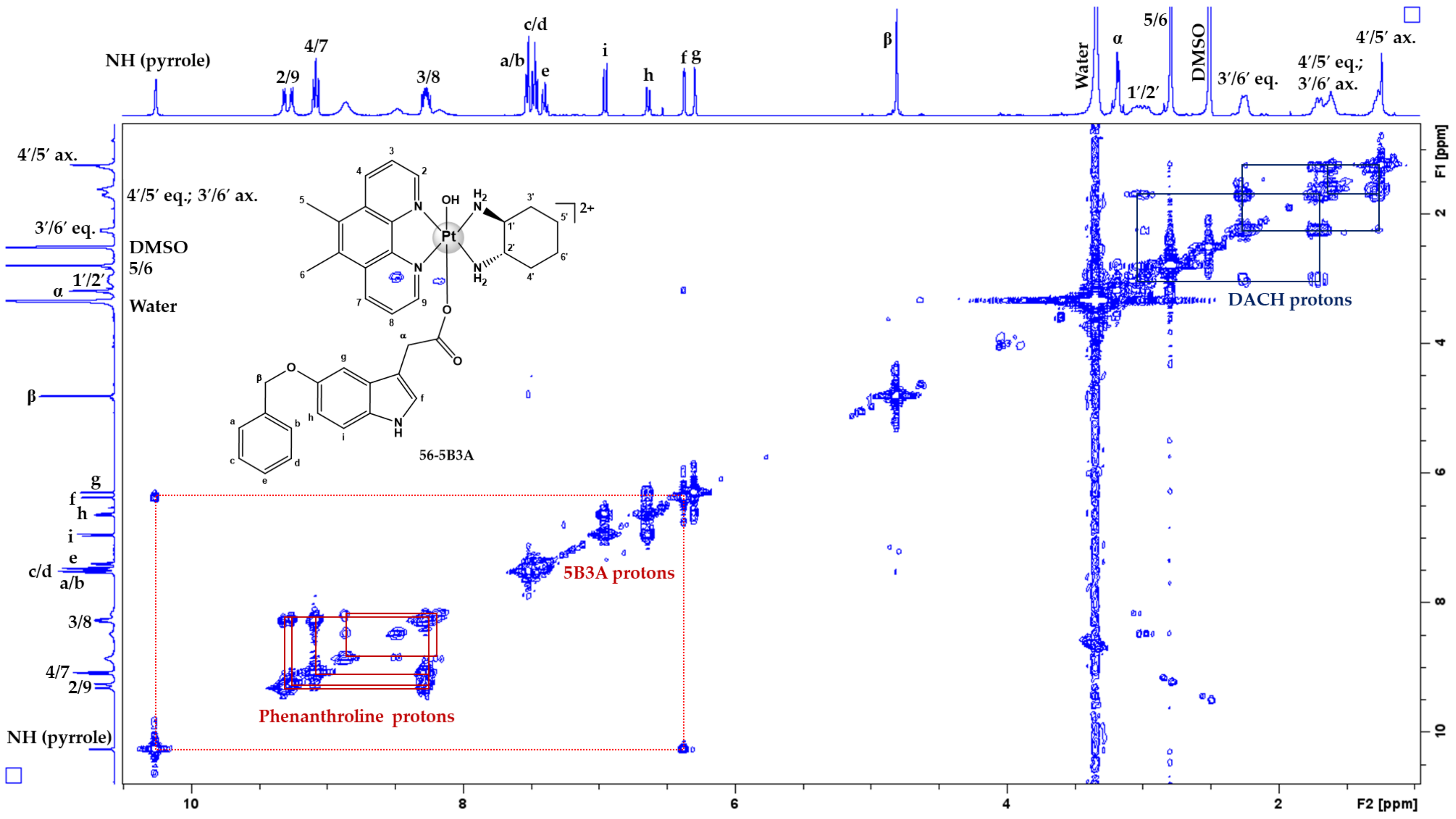

Figure S14: 2D-COSY spectrum of **56-5B3A** in DMSO-d6.

^1H-^{195}Pt-HMQC Spectra of P-5B3A, 5-5B3A and 56-5B3A

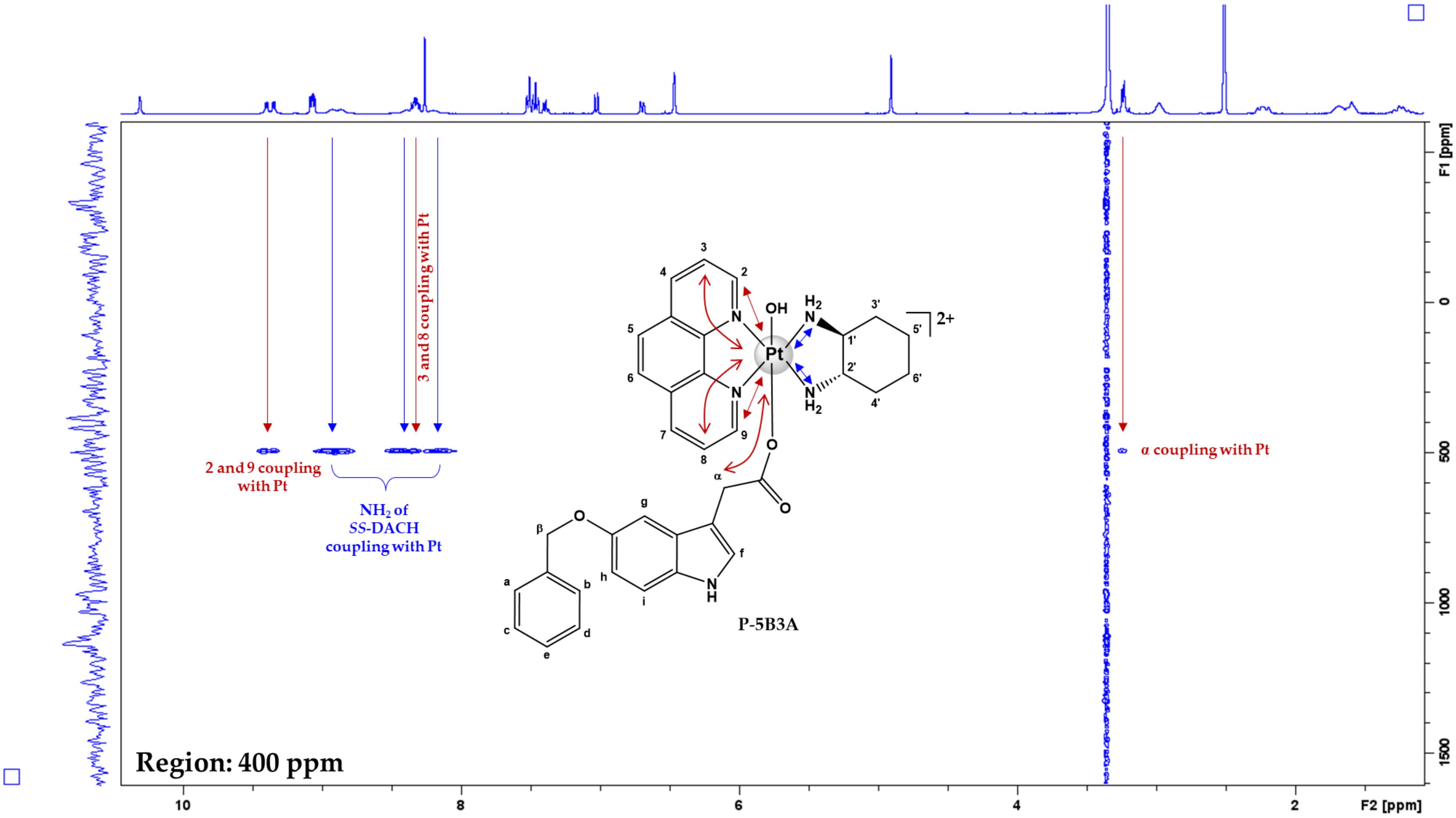

Figure S15: ^1H-^{195}Pt-HMQC spectrum of **P-5B3A** in DMSO-d_6 at 400 ppm.

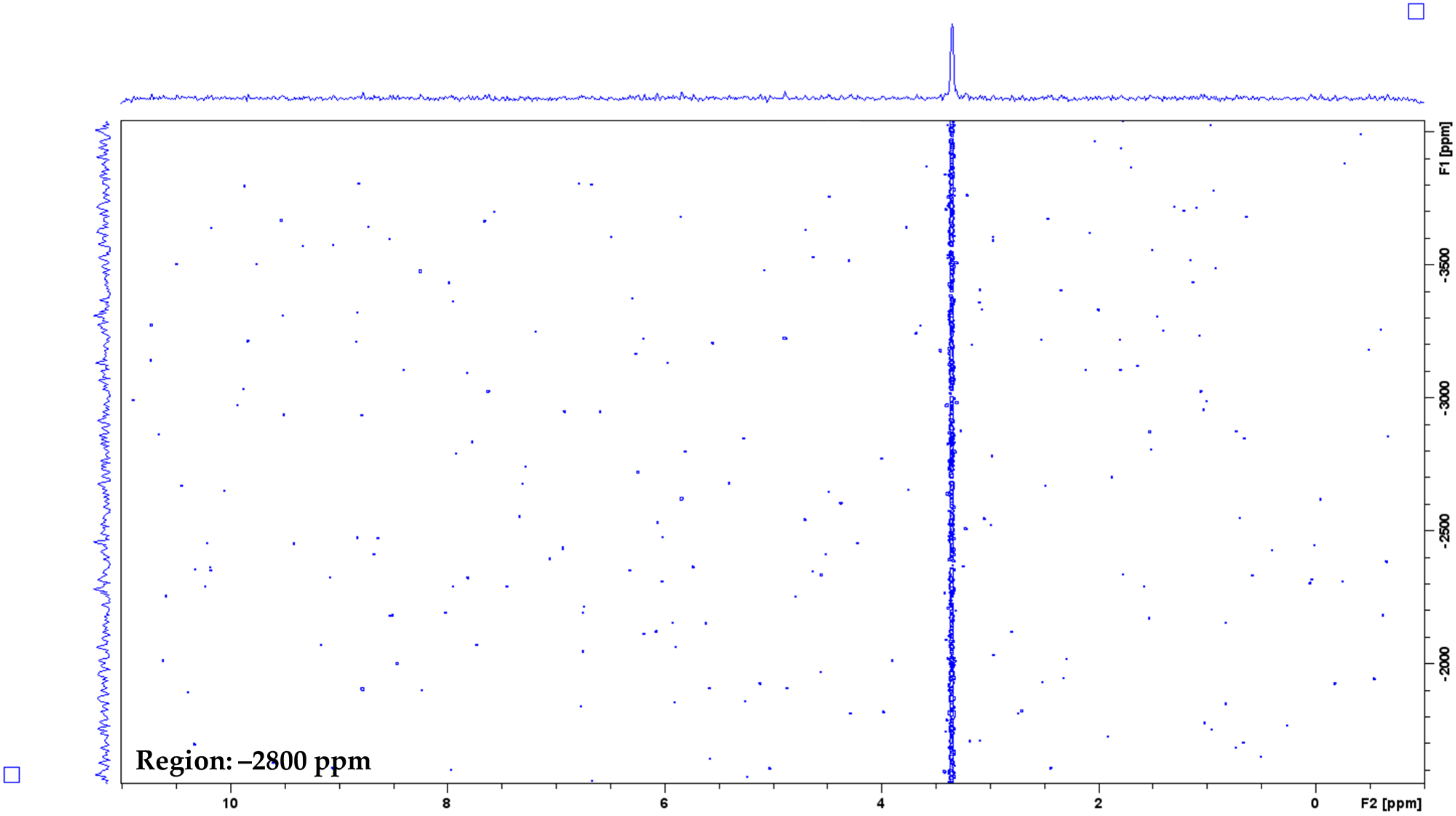

Figure S16: ^{1}H-^{195}Pt-HMQC spectrum of **P-5B3A** in DMSO-d_6 at –2800 ppm.

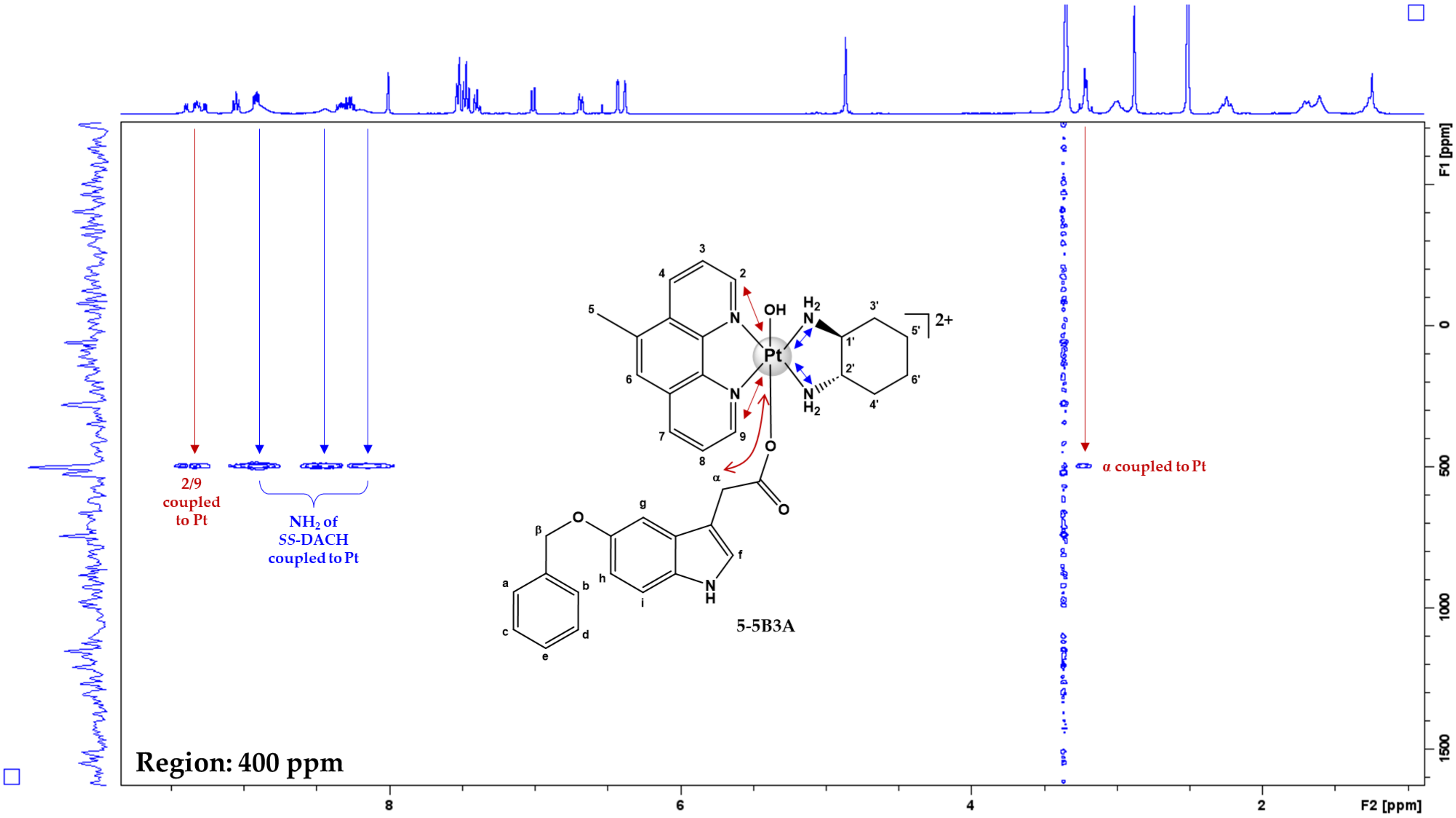

Figure S17: ^{1}H-^{195}Pt-HMQC spectrum of **5-5B3A** in DMSO-d6 at 400 ppm.

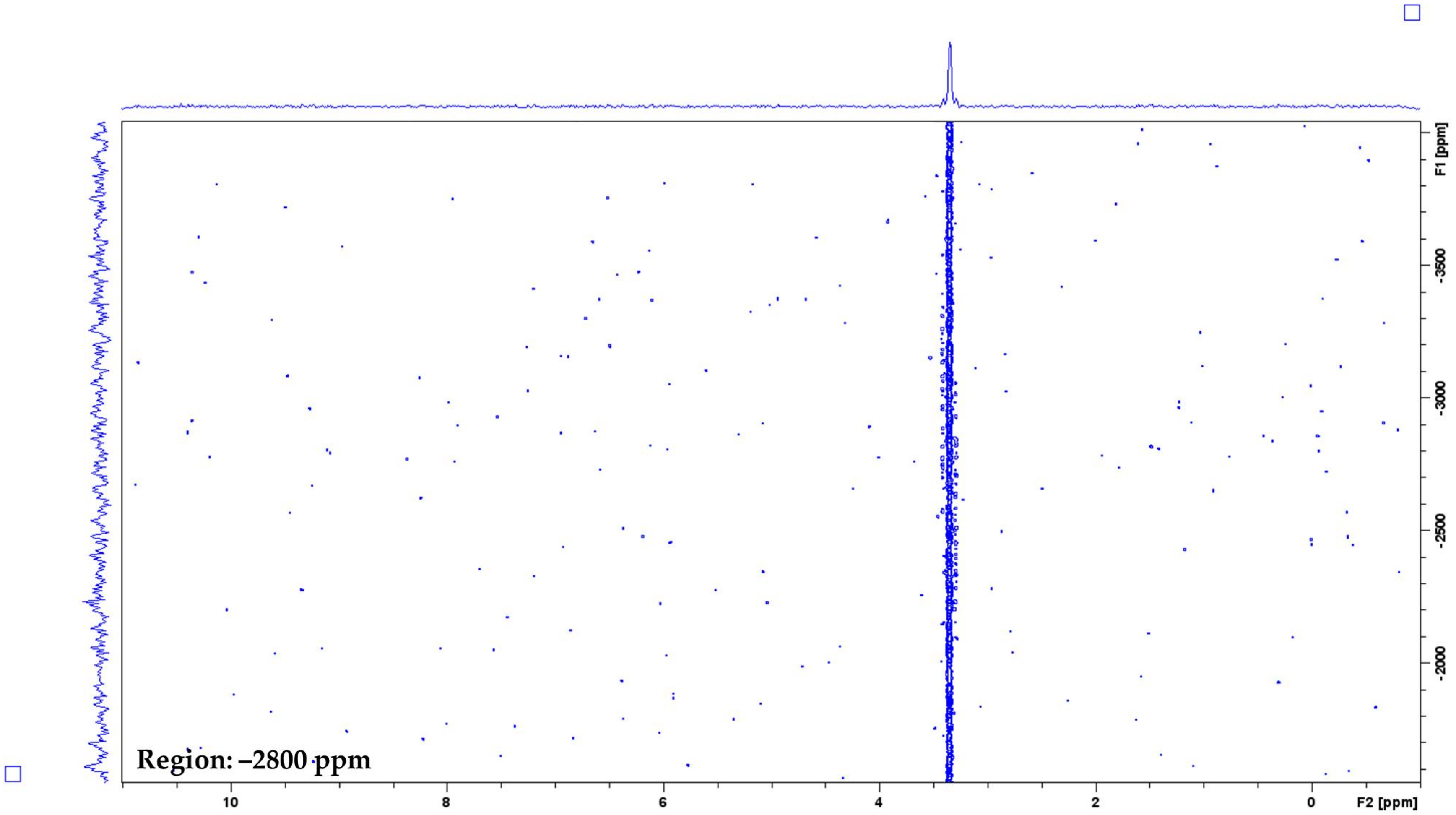

Figure S18: ^{1}H-^{195}Pt-HMQC spectrum of **5-5B3A** in DMSO-d_6 at –2800 ppm.

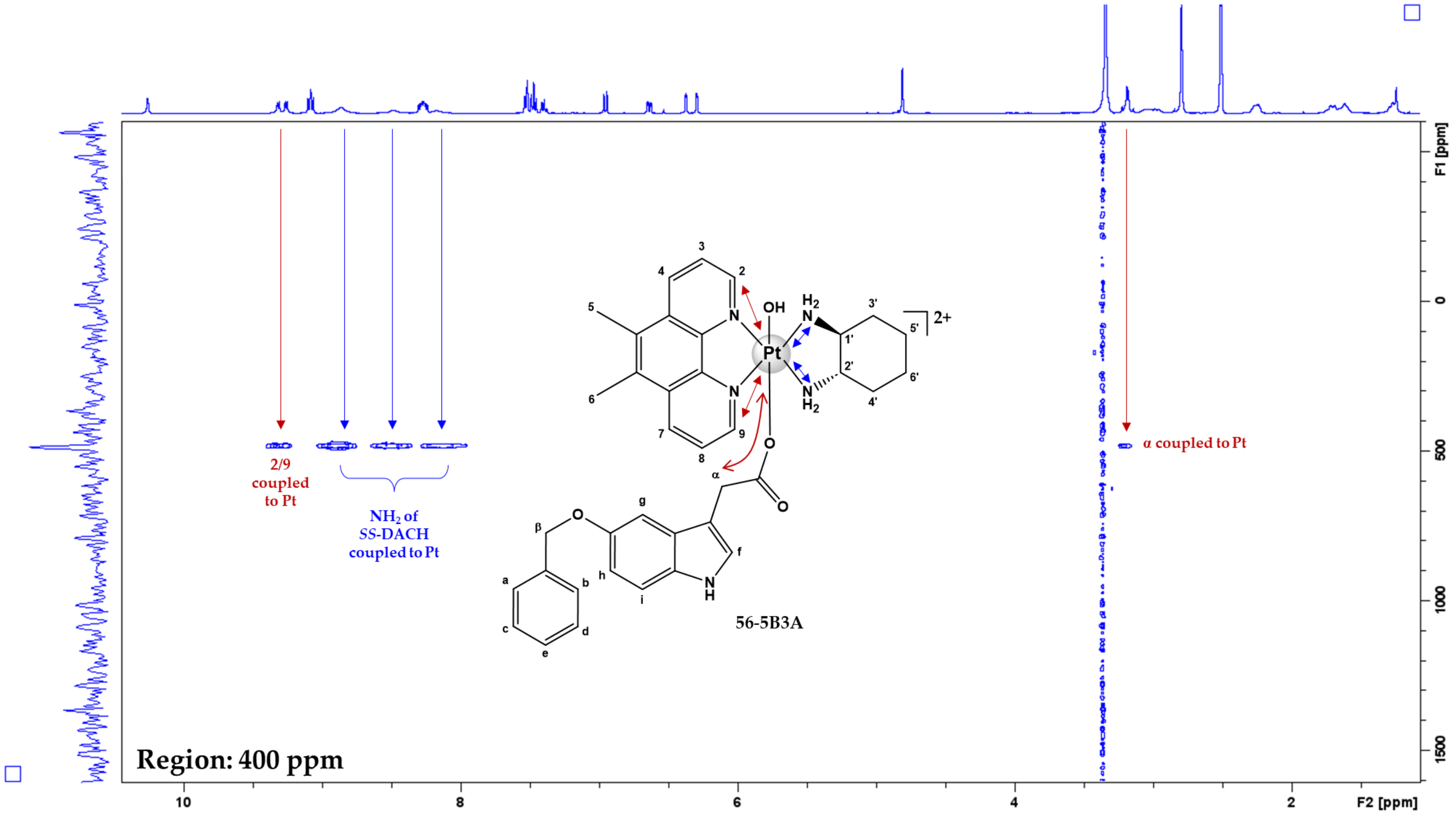

Figure S19: ^{1}H-^{195}Pt-HMQC spectrum of **56-5B3A** in DMSO-d_6 at 400 ppm.

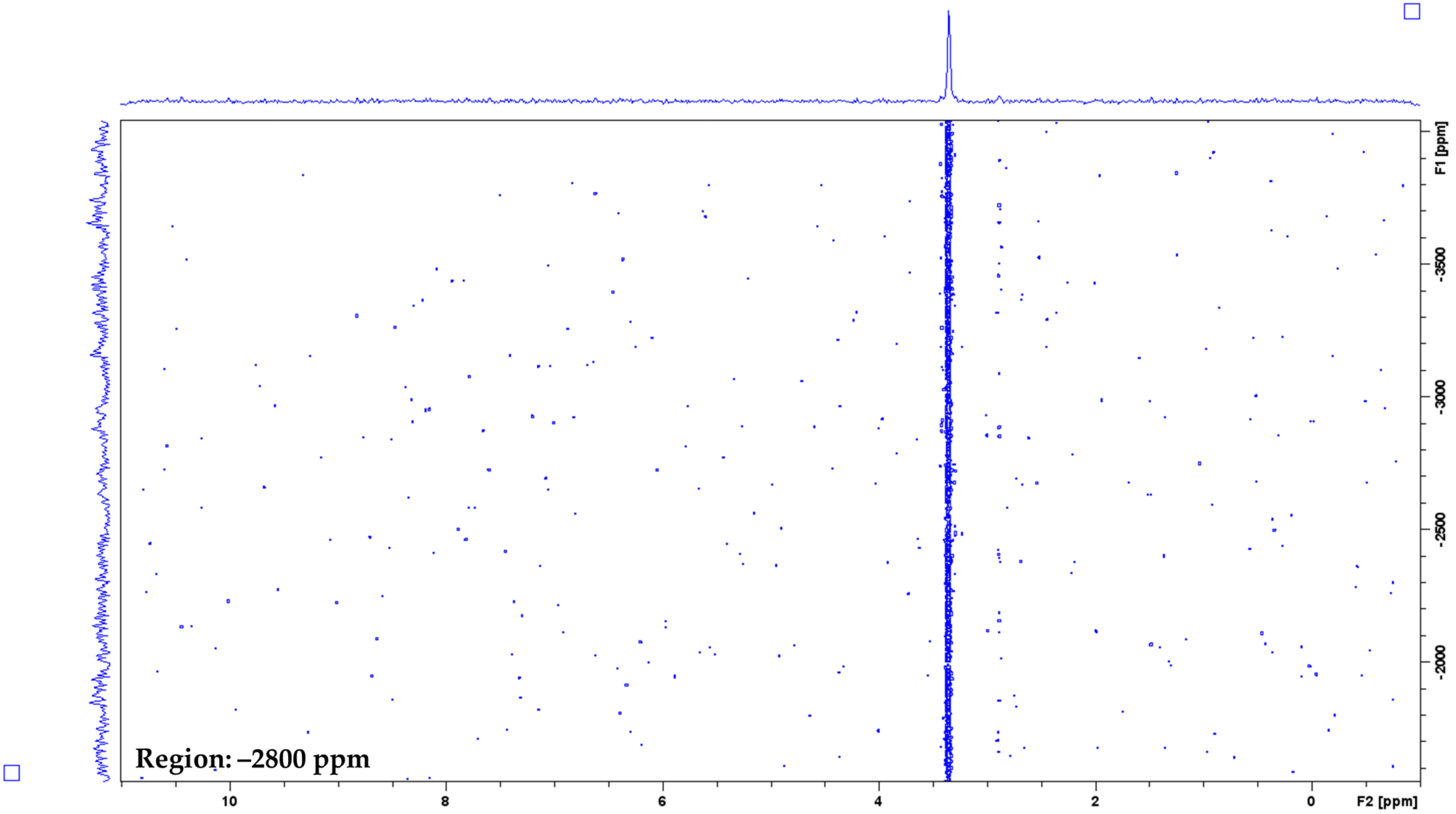

Figure S20: ^{1}H-^{195}Pt-HMQC spectrum of **56-5B3A** in DMSO-d_6 at –2800 ppm.

UV Spectra of P-5B3A, 5-5B3A and 56-5B3A

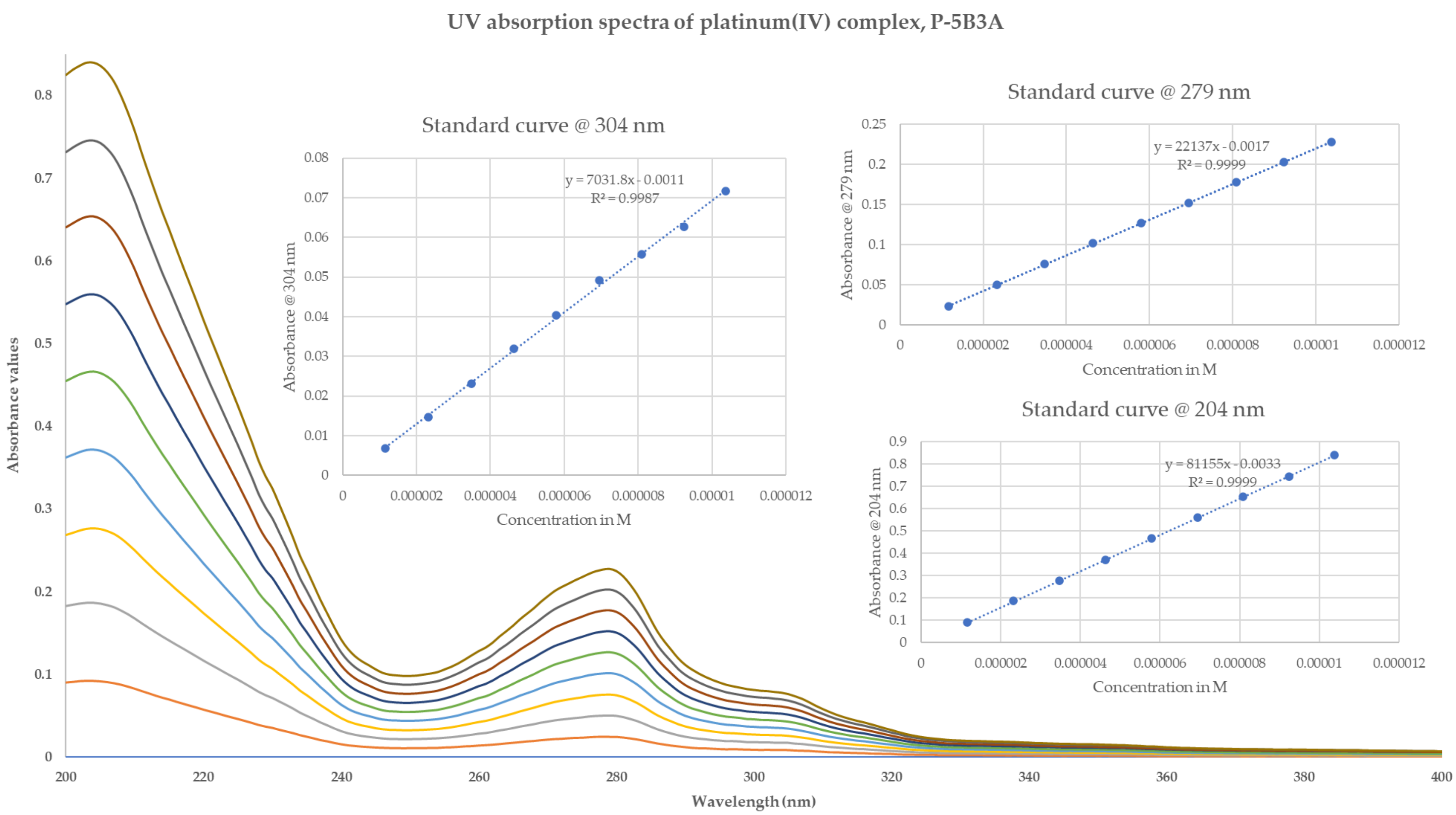

Figure S21: UV spectra of **P-5B3A** and plot curves at 204, 279 and 304 nm.

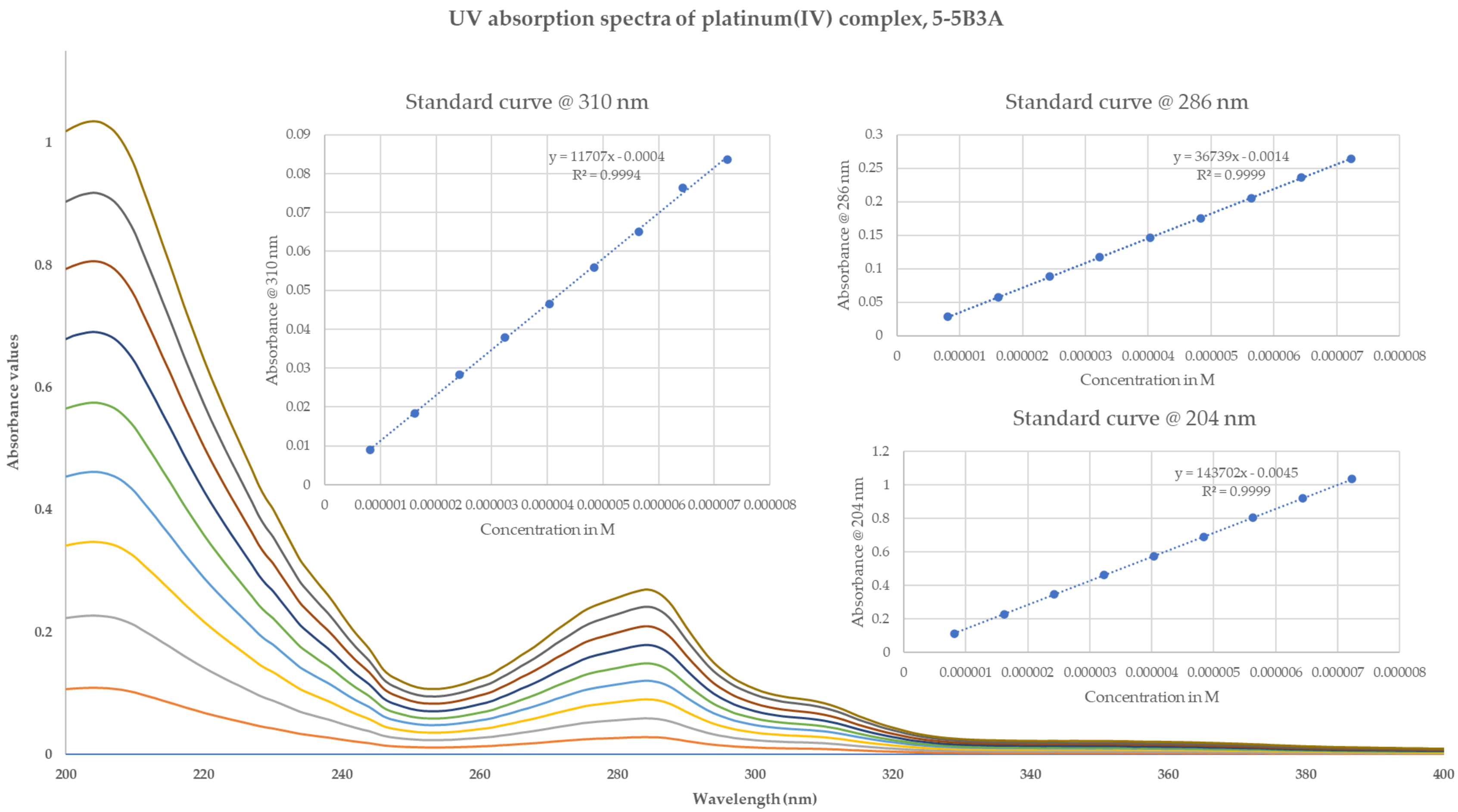

Figure S22: UV spectra of **5-5B3A** and plot curves at 204, 286 and 310 nm.

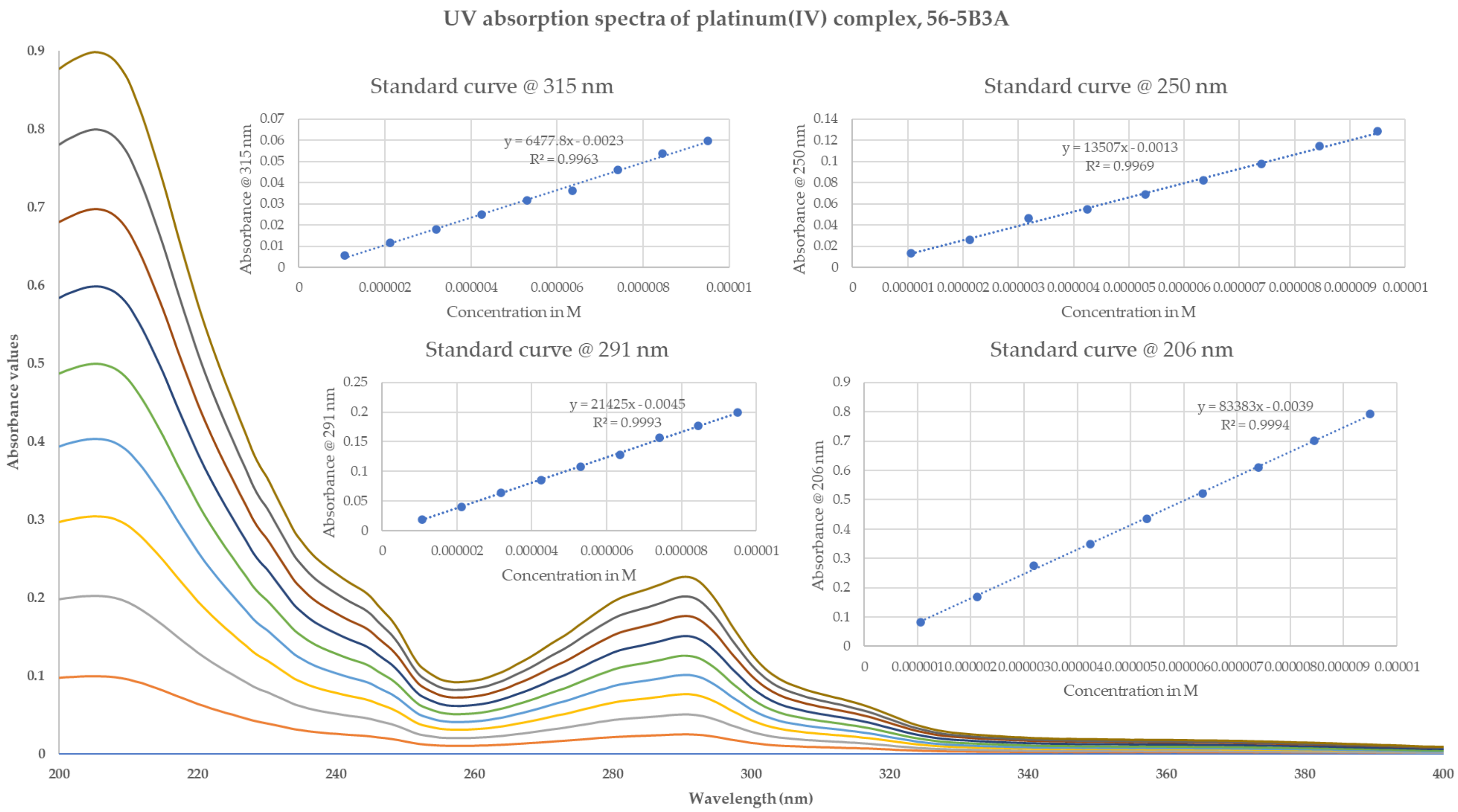

Figure S23: UV spectra of **56-5B3A** and plot curves at 206, 250, 291 and 315 nm.

CD Spectra of P-5B3A, 5-5B3A and 56-5B3A

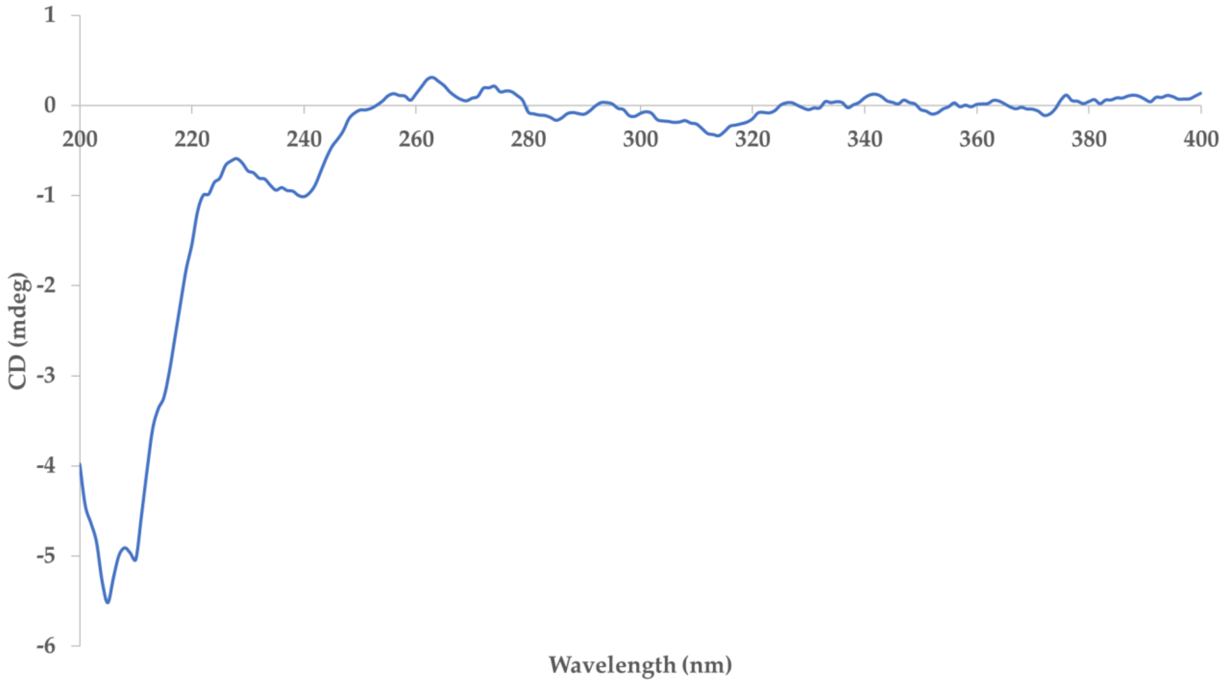

Figure S24: CD spectrum of **P-5B3A** (0.05 mM) acquired after 30 accumulations.

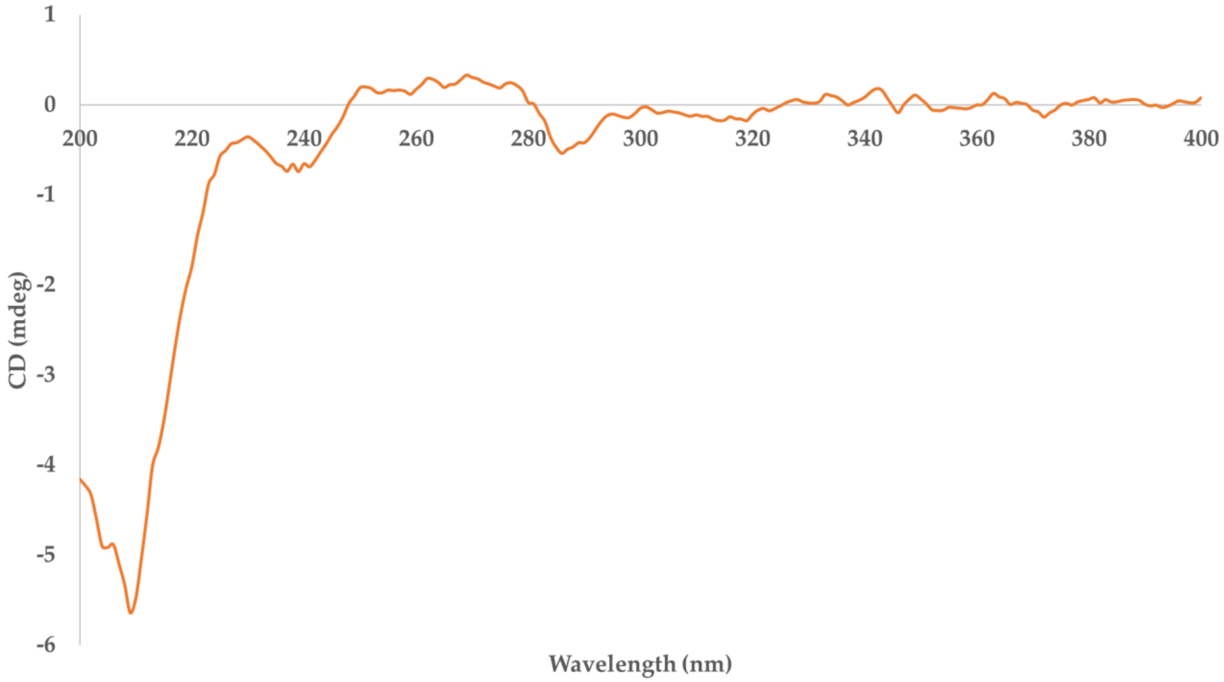

Figure S25: CD spectrum of **5-5B3A** (0.05 mM) acquired after 30 accumulations.

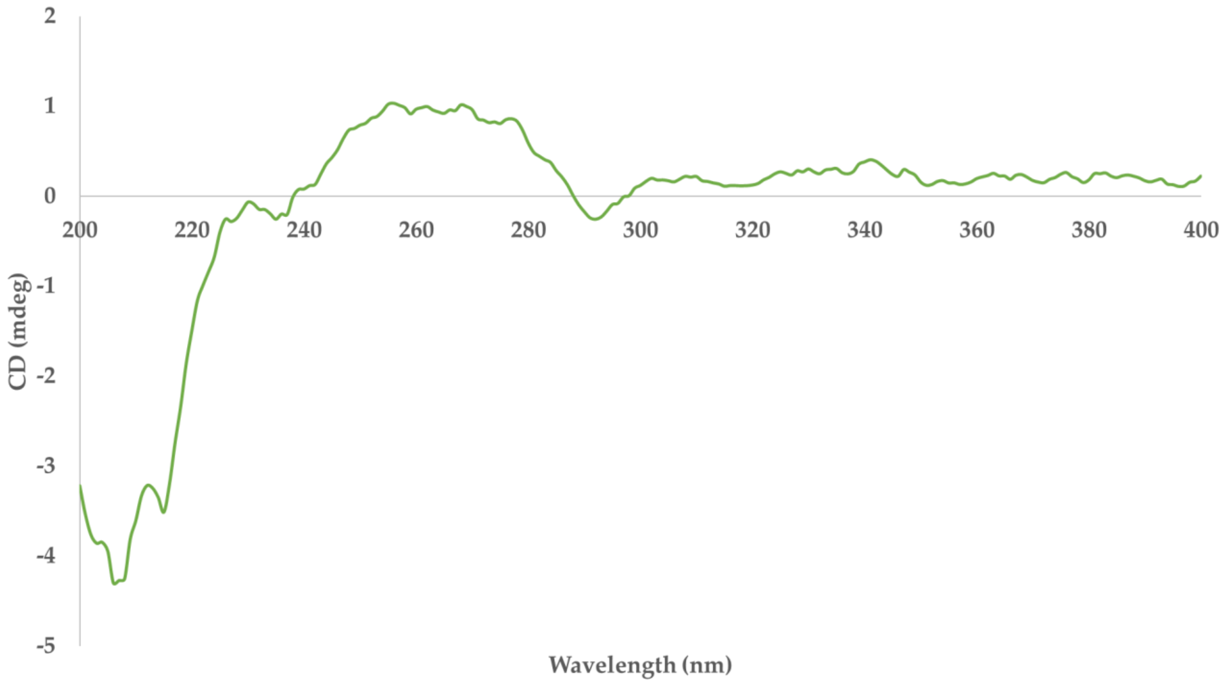

Figure S26: CD spectrum of **56-5B3A** (0.05 mM) acquired after 30 accumulations.

Lipophilicity Measurements

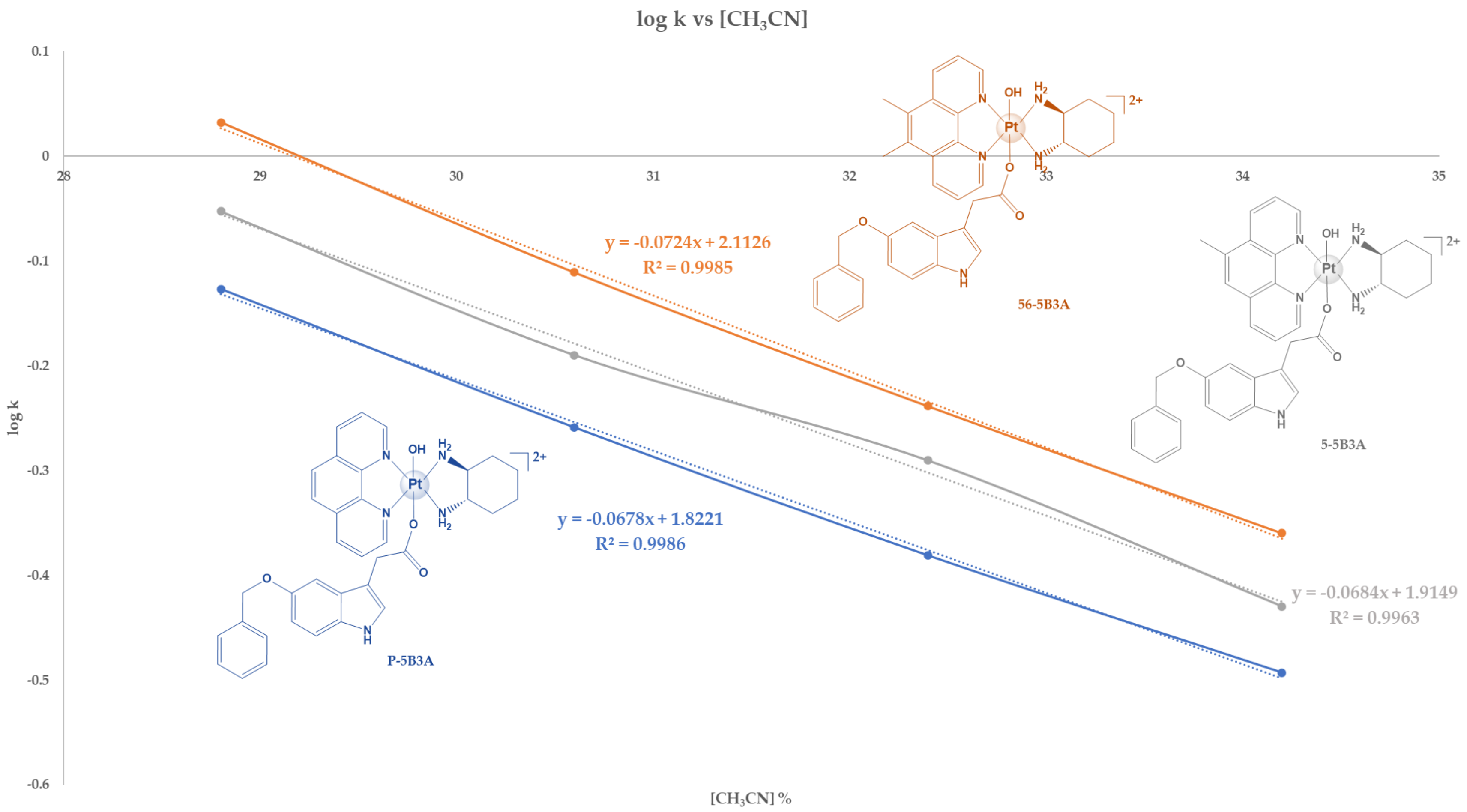

Figure S27: Plots of log k versus concentration of the organic solvent, CH_3CN to determine the chromatographic lipophilicity index, log k_w of **P-5B3A**, **5-5B3A** and **56-5B3A**.

Reduction Measurements for P-5B3A, 5-5B3A and 56-5B3A

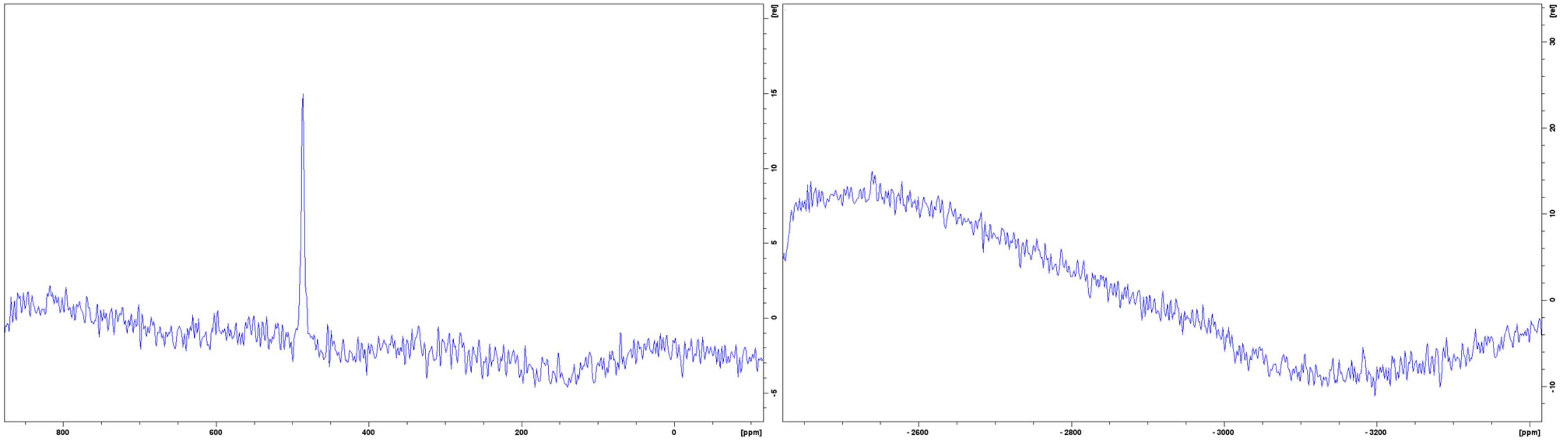

Region: 400 ppm

T = 0

Region: –2800 ppm

T = 1 h

Figure S28: Preliminary 1D-^{195}Pt-NMR spectra of **P-5B3A** with PBS in DMSO-d_6 within the regions of 400 and –2800 ppm and at 37 °C.

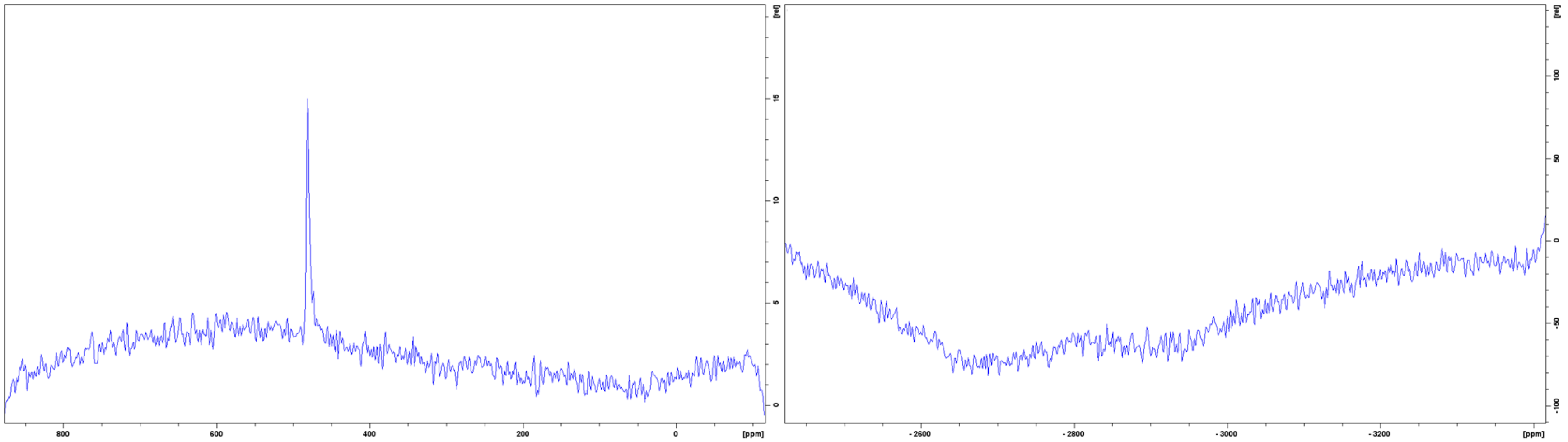

Region: 400 ppm

T = 0

Region: –2800 ppm

T = 1 h

Figure S29: Preliminary 1D-^{195}Pt-NMR spectra of **5-5B3A** with PBS in DMSO-d_6 within the regions of 400 and –2800 ppm and at 37 °C.

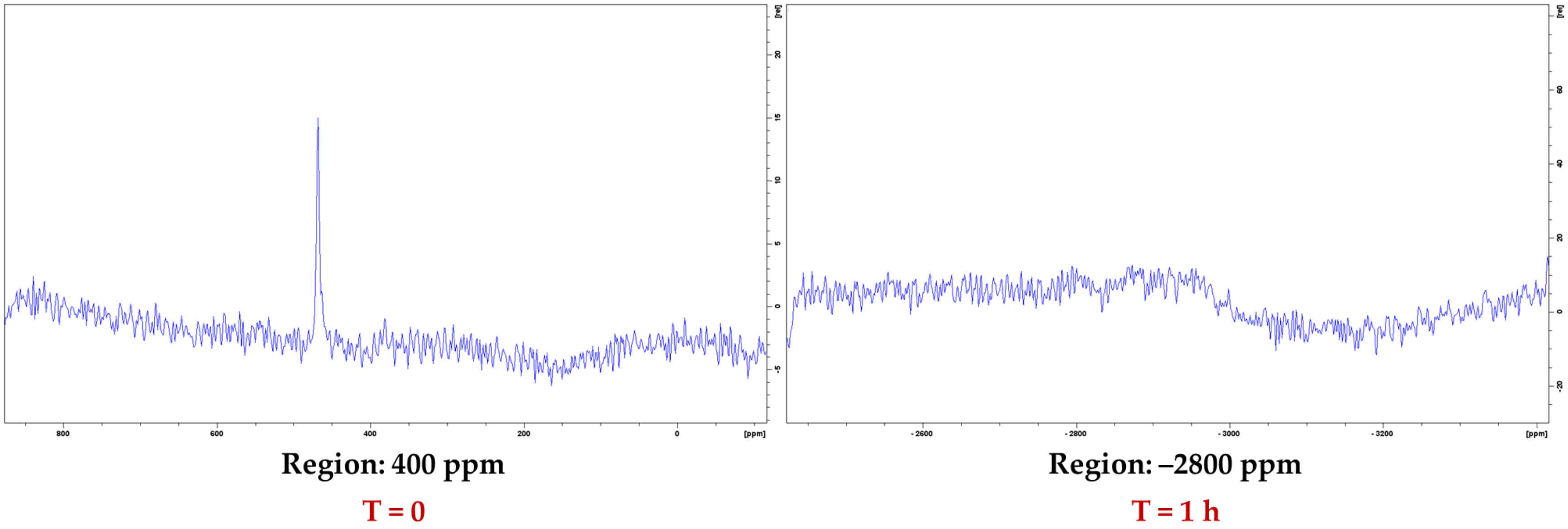

Figure S30: Preliminary 1D-^{195}Pt-NMR spectra of **56-5B3A** with PBS in DMSO-d_6 within the regions of 400 and –2800 ppm and at 37 °C.

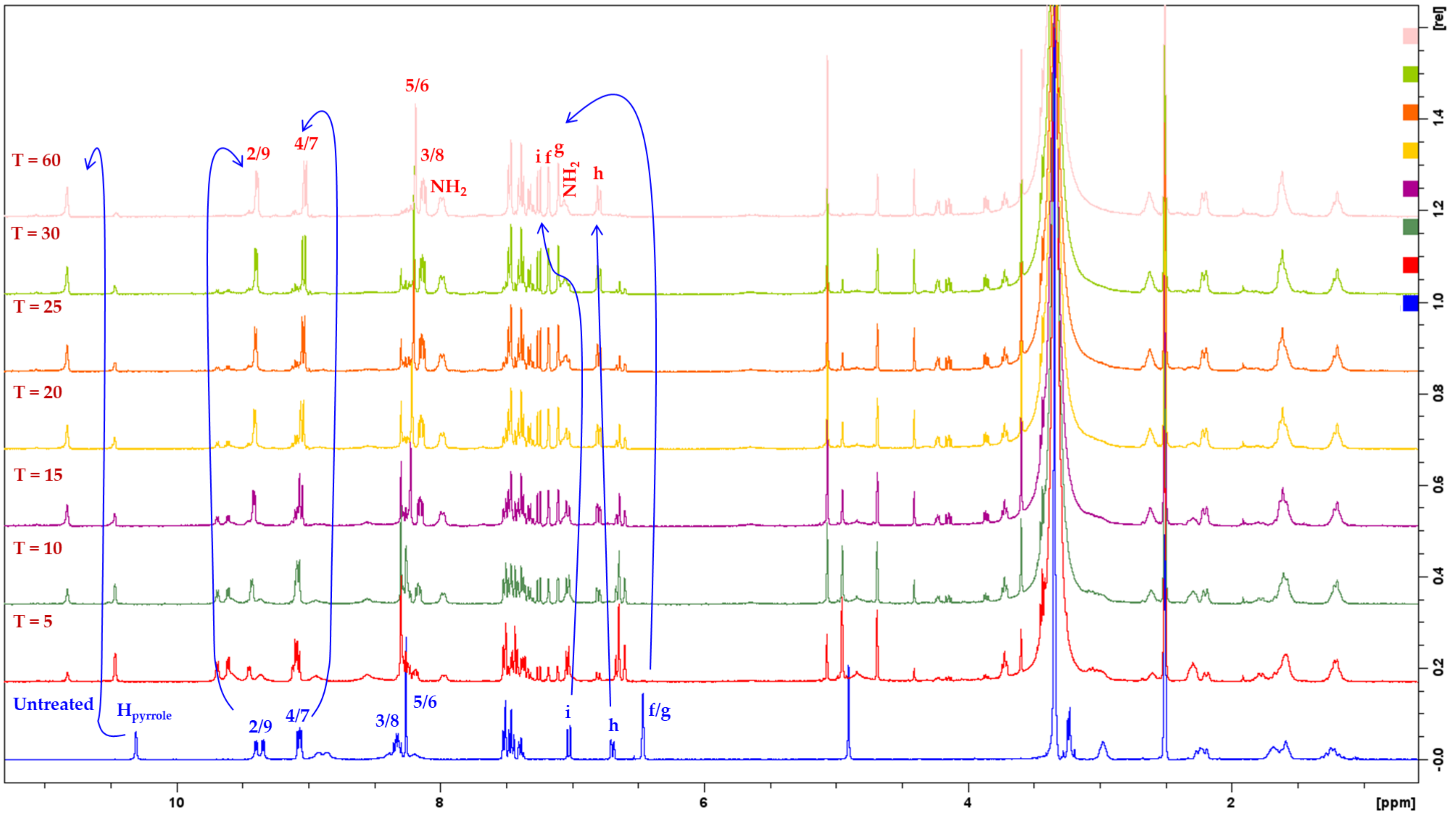

Figure S31: ^{1}H-NMR spectra of **P-5B3A** with PBS and AsA in DMSO-d$_6$ at 37 °C, in different time intervals, displaying the shift in chemical multiplicity. **T** is time in min.

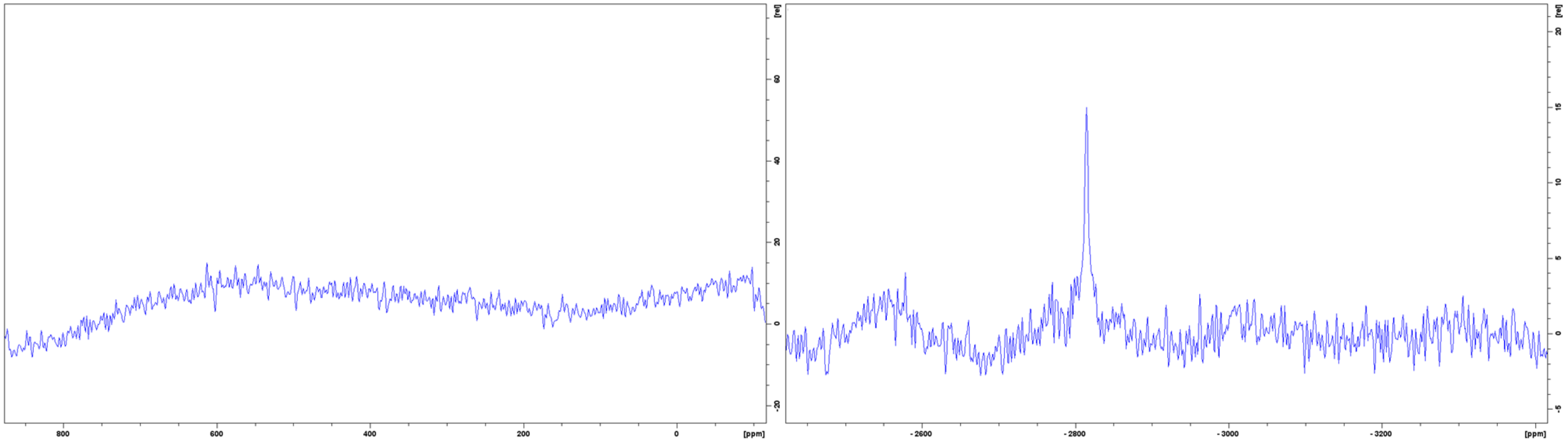

Region: 400 ppm

T = 1.5 h

Region: –2800 ppm

T = 2 h

Figure S32: 1D-^{195}Pt-NMR spectra of **P-5B3A** with PBS and AsA in DMSO-d_6, within the regions of 400 and –2800 ppm at 37 °C, displaying its complete reduction after 2 h from the final ^{1}H-NMR experiment.

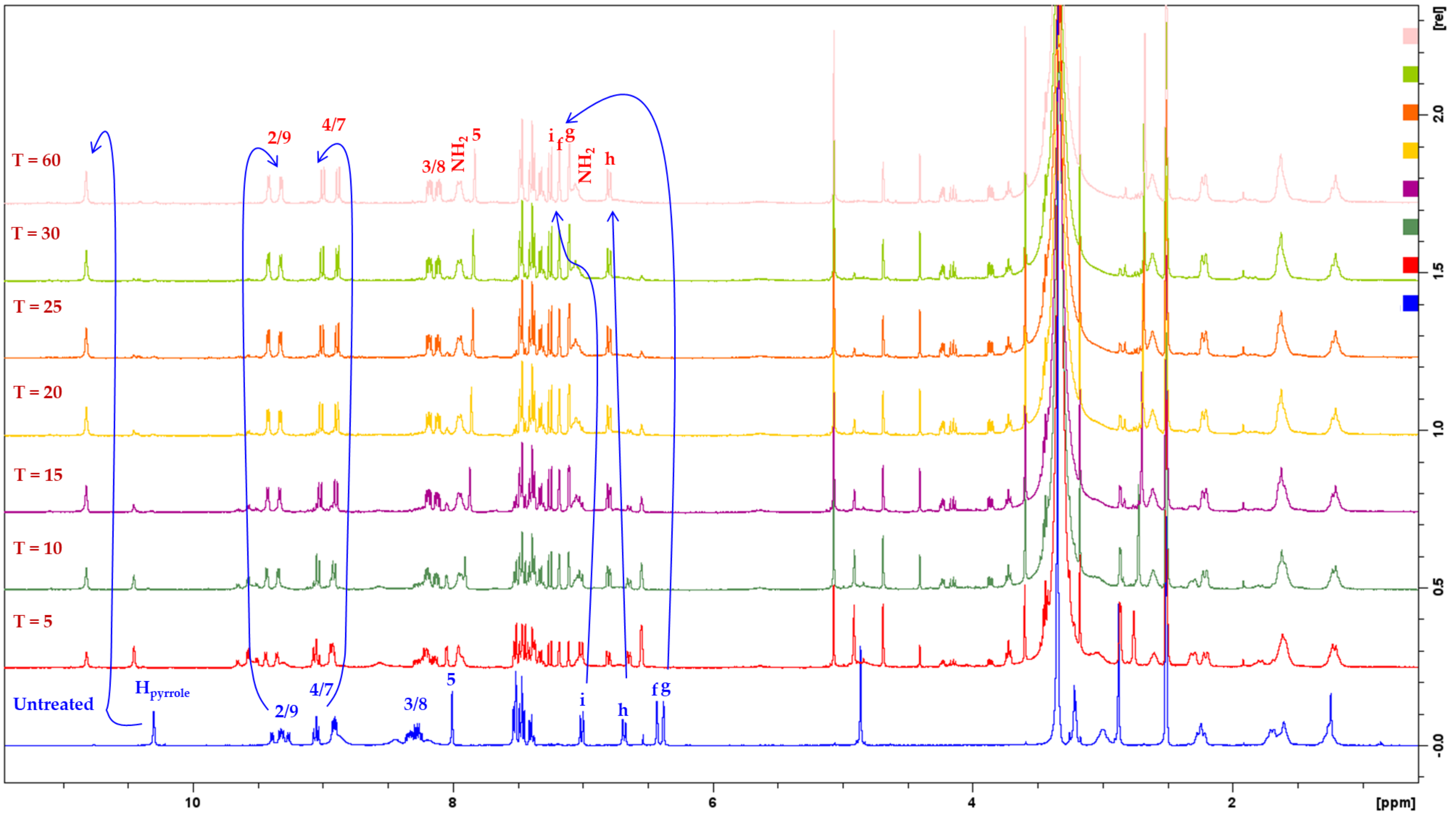

Figure S33: ^{1}H-NMR spectra of **5-5B3A** with PBS and AsA in DMSO-d$_6$ at 37 °C, in different time intervals, displaying the shift in chemical multiplicity. **T** is time in min.

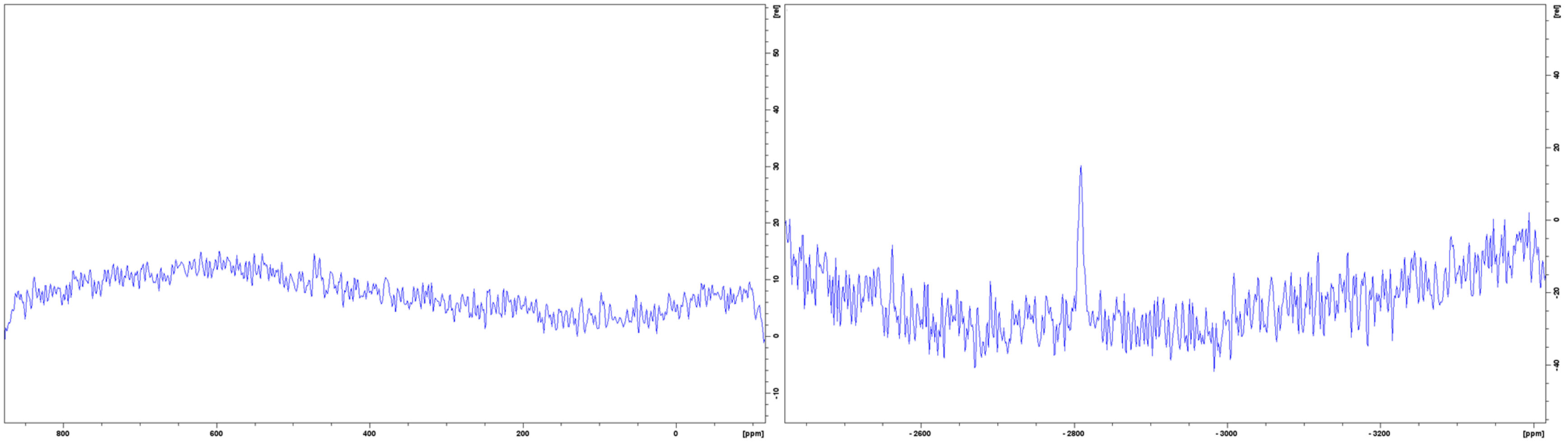

Region: 400 ppm

T = 1.5 h

Region: –2800 ppm

T = 2 h

Figure S34: 1D-^{195}Pt-NMR spectra of **5-5B3A** with PBS and AsA in DMSO-d_6, within the regions of 400 and –2800 ppm at 37 °C, displaying its complete reduction after 2 h from the final ^{1}H-NMR experiment.

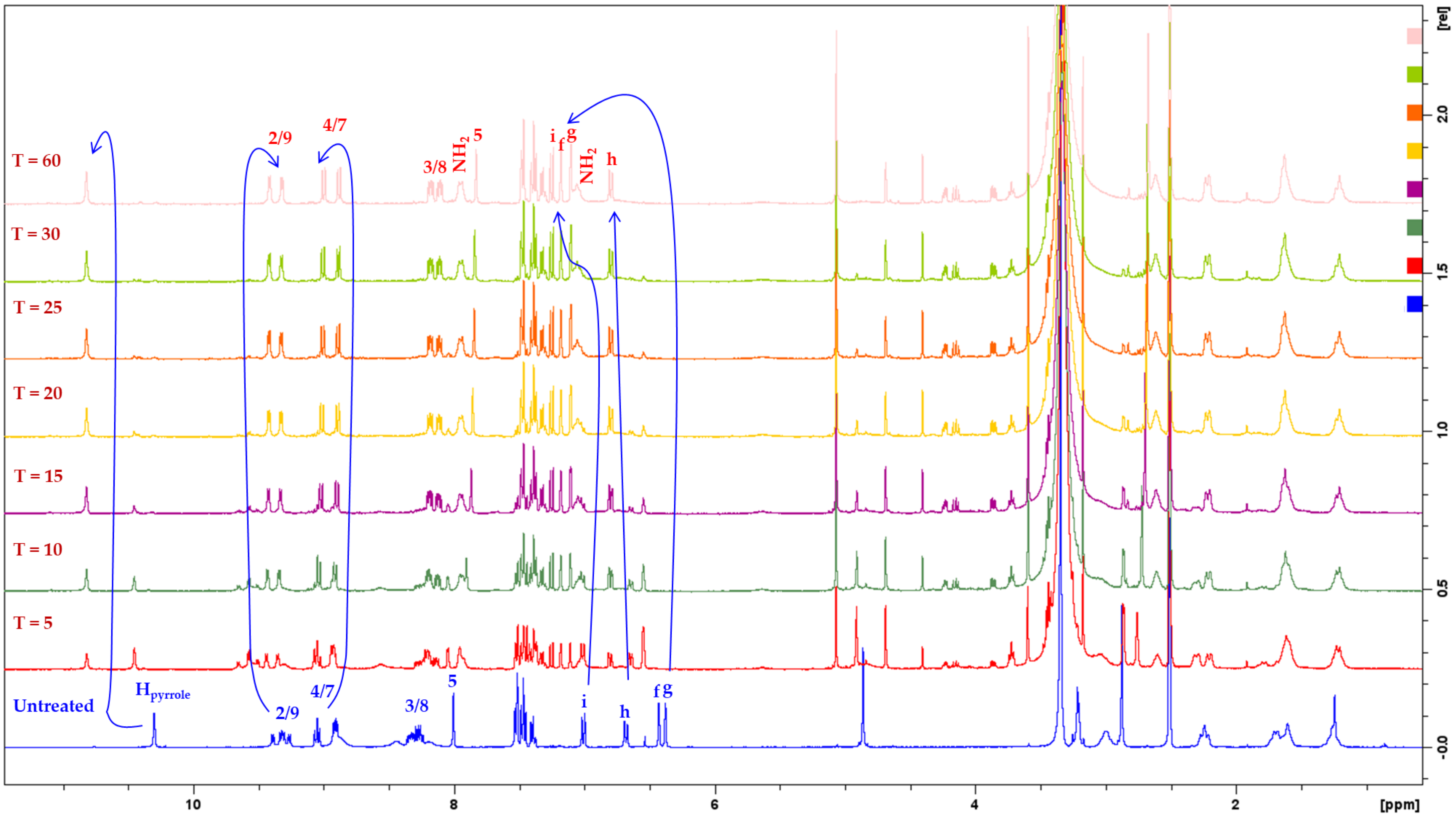

Figure S35: ^{1}H-NMR spectra of **56-5B3A** with PBS and AsA in DMSO-d_6 at 37 °C, in different time intervals, displaying the shift in chemical multiplicity. **T** is time in min.

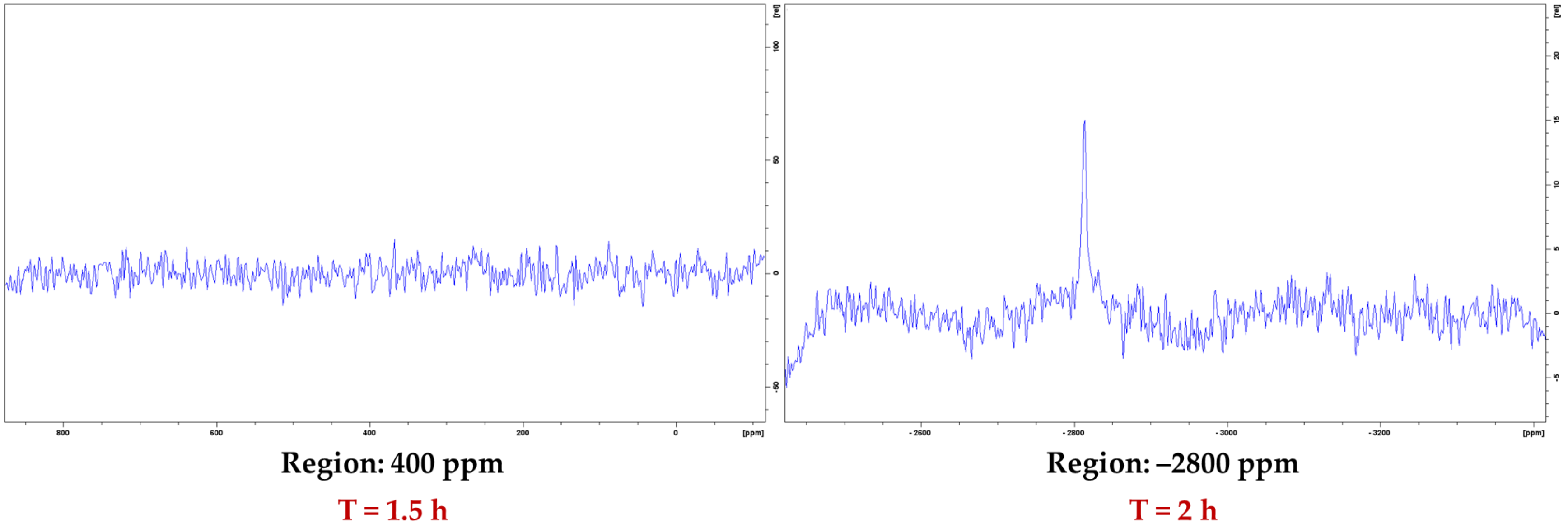

Figure S36: 1D-^{195}Pt-NMR spectra of **56-5B3A** with PBS and AsA in DMSO-d_6, within the regions of 400 and –2800 ppm at 37 °C, displaying its complete reduction after 1 h from the final ^{1}H-NMR experiment.

Stability Measurements for P-5B3A, 5-5B3A and 56-5B3A in PBS at 37 °C

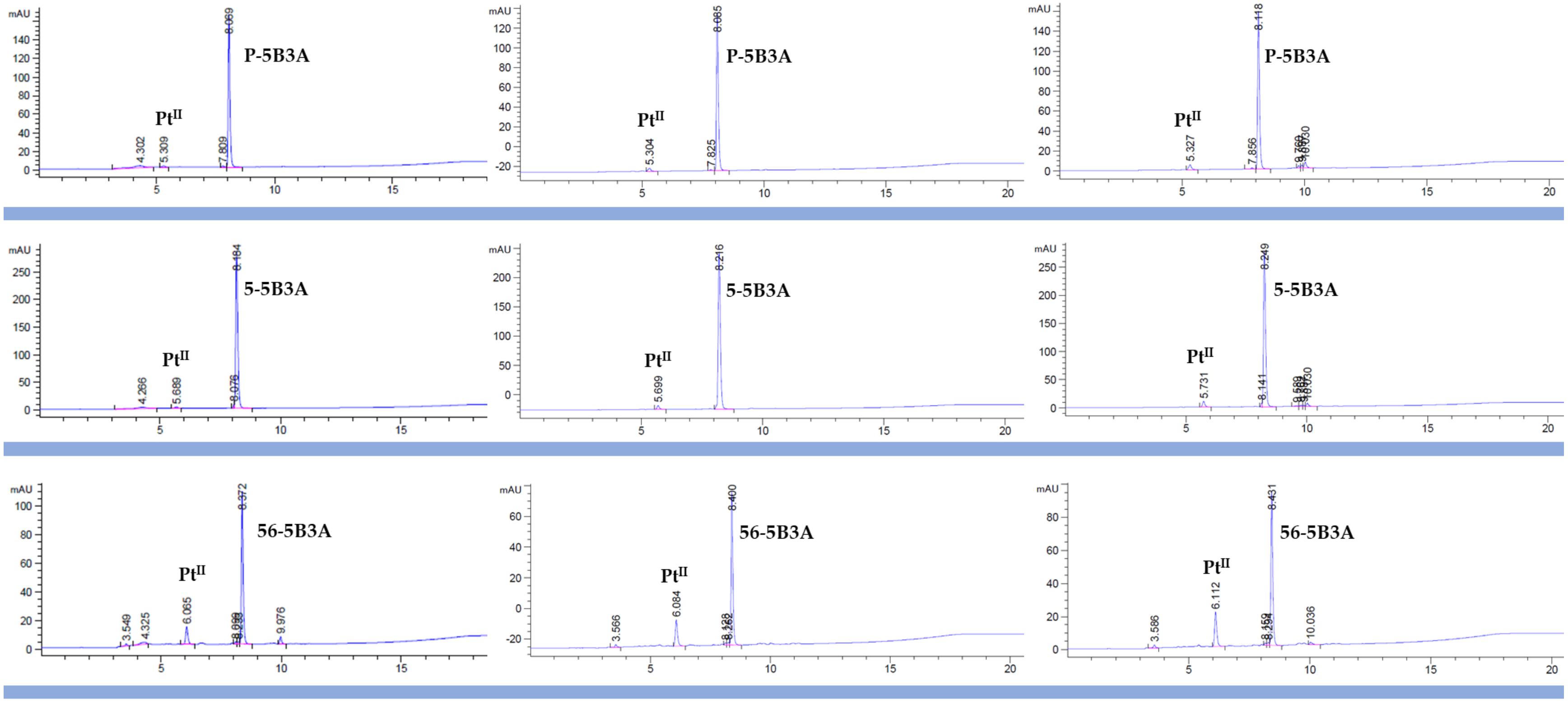

A: HPLC Chromatograms (0 h) **A: HPLC Chromatograms (36 h)** **B: HPLC Chromatograms (72 h)**

Figure S37: Combined HPLC chromatograms acquired for **P-5B3A**, **5-5B3A** and **56-5B3A** at 254 nm after treatment with PBS at 37 °C, at 0, 36 and 72 h.

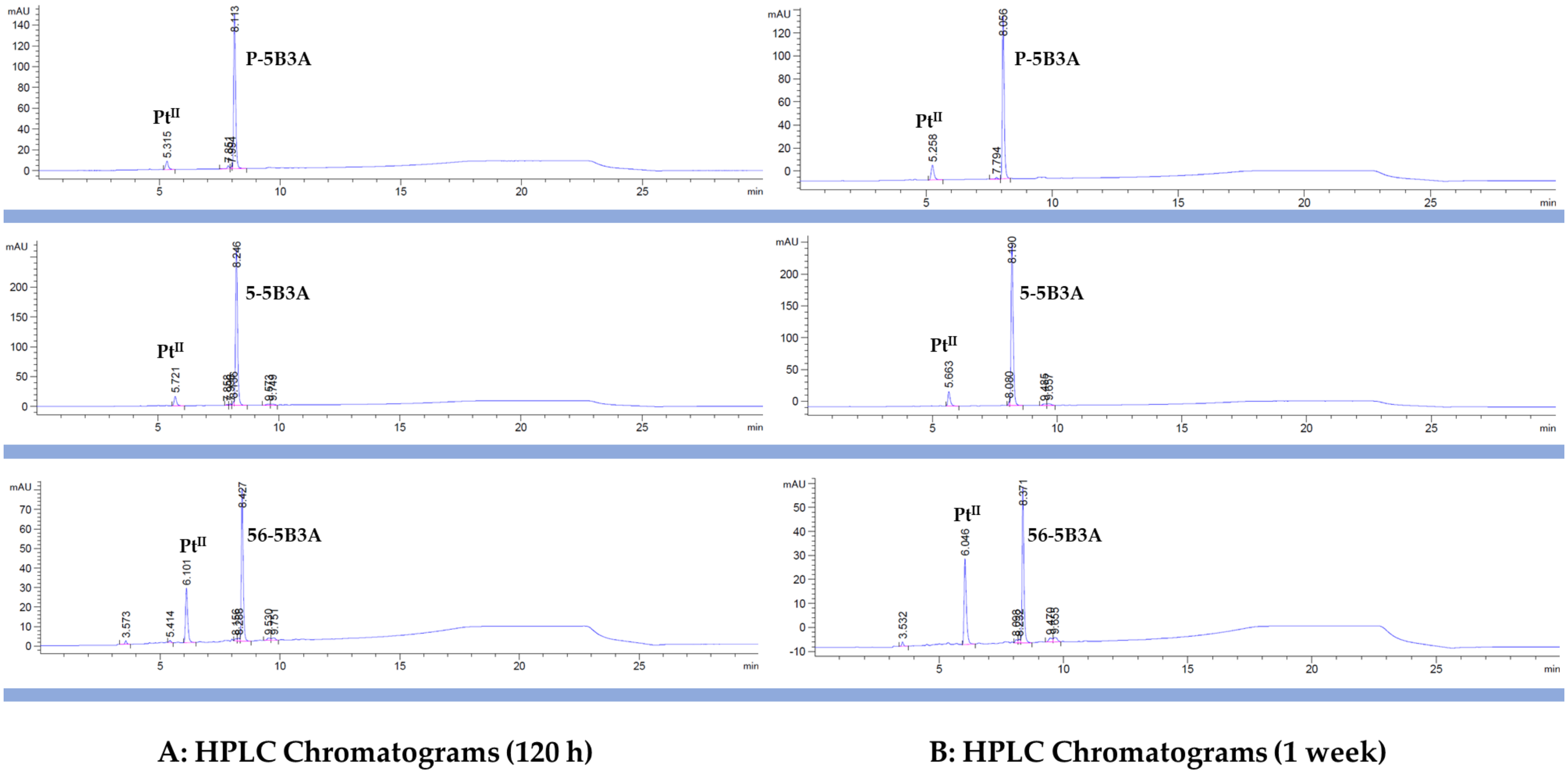

Figure S38: Combined HPLC chromatograms acquired for **P-5B3A**, **5-5B3A** and **56-5B3A** at 254 nm after treatment with PBS at 37 °C, at 120 h and 1 week.

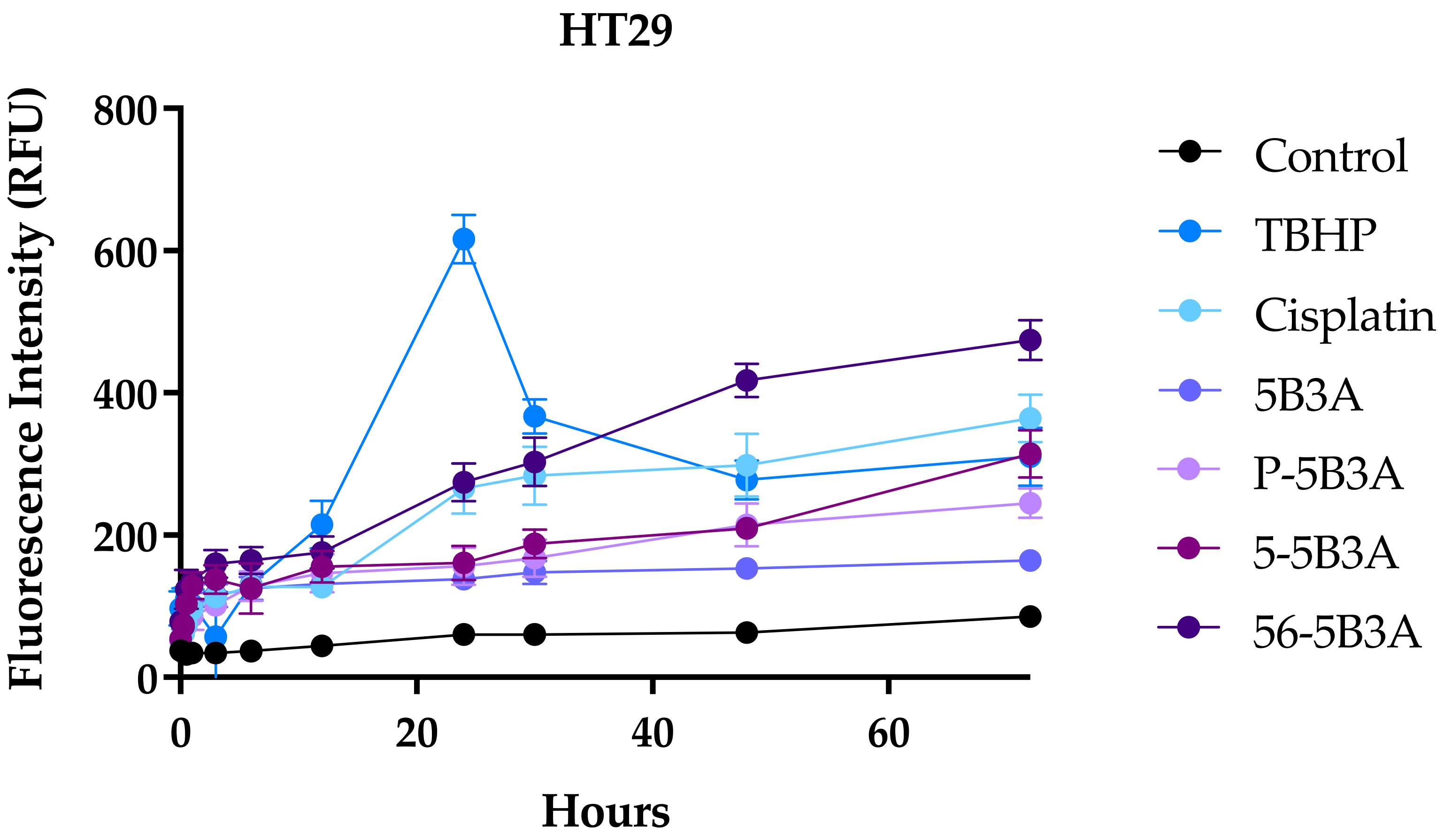

Figure S39: ROS production upon treatment with **P-5B3A**, **5-5B3A** and **56-5B3A**, and cisplatin in HT29 colon cells at 0, 0.25, 0.5, 1, 3, 6, 12, 24, 48 and 72 h. TBHP: tert-butyl hydroperoxide. Data points denote mean ± SEM. $n = 3$ from three independent experiments where samples were run in triplicates.

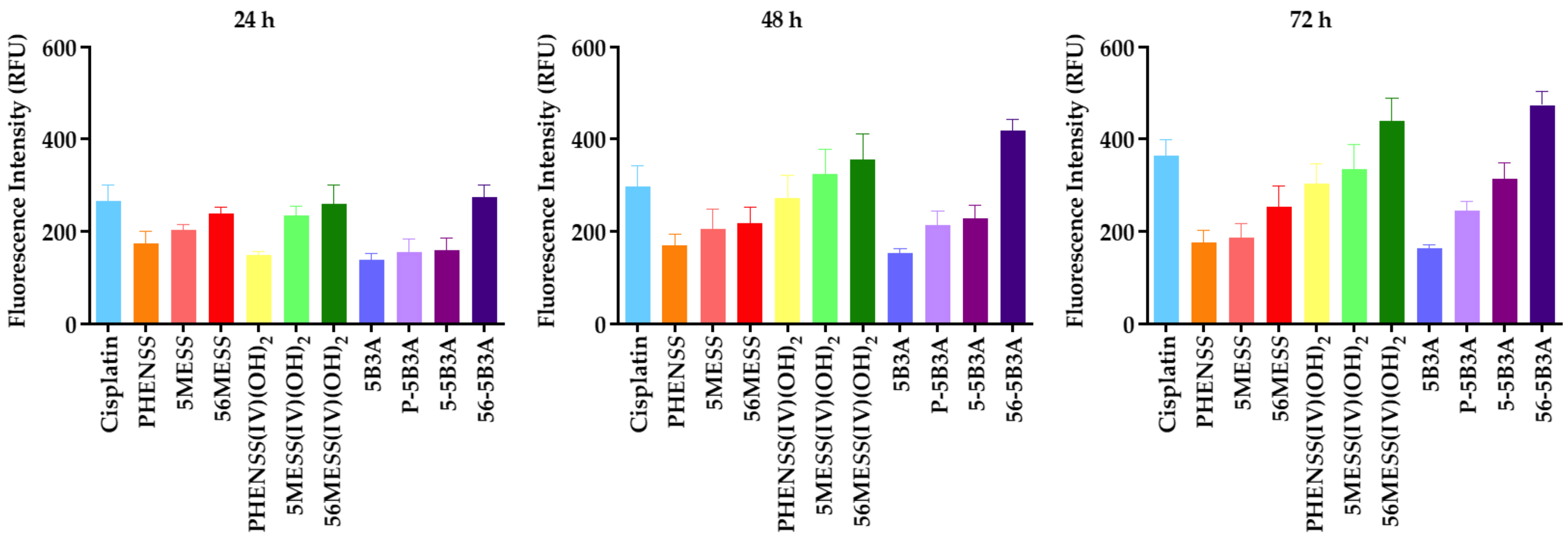

Figure S40: ROS generation in HT29 colon cells after treatment with **P-5B3A**, **5-5B3A** and **56-5B3A**, 5B3A, the equivalent platinum(II) precursors (**PHEN*SS***, **5ME*SS*** and **56ME*SS***) and platinum(IV) scaffolds (**PHEN*SS*(IV)(OH)$_2$**, **5ME*SS*(IV)(OH)$_2$** and **56ME*SS*(IV)(OH)$_2$**), and cisplatin at 24, 48 and 72 h. Data are expressed in RFU values. The RFU values of the platinum(II) precursors (**PHEN*SS***, **5ME*SS*** and **56ME*SS***) and the platinum(IV) scaffolds (**PHEN*SS*(IV)(OH)$_2$**, **5ME*SS*(IV)(OH)$_2$** and **56ME*SS*(IV)(OH)$_2$**) were included in the diagrams for comparison. Data points signify mean ± SEM. n = 3 from three independent tests where samples were achieved in triplicate. Significance was identified by Tukey's multiple comparisons test for multiple comparisons compared with cisplatin, as measured by one-way ANOVA.

MtMP Measurements

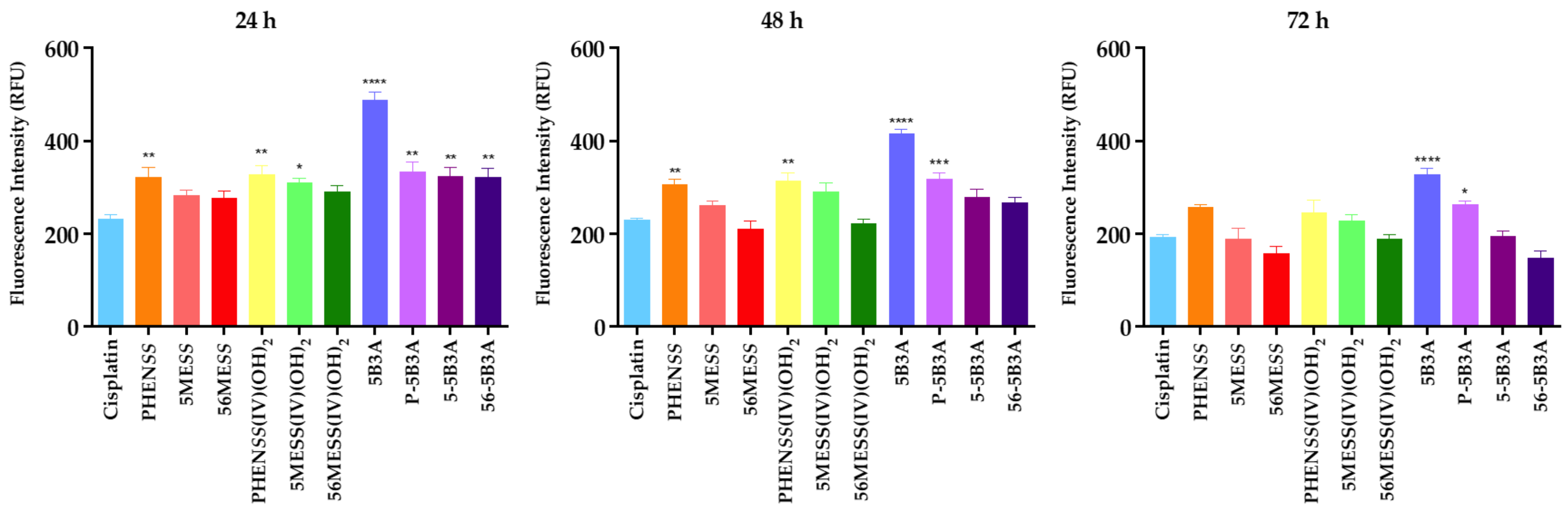

Figure S41: The detected MtMP changes in HT29 colon cells after treatment with **P-5B3A**, **5-5B3A** and **56-5B3A**, 5B3A, the equivalent platinum(II) precursors (**PHEN*SS***, **5ME*SS*** and **56ME*SS***) and platinum(IV) scaffolds (**PHEN*SS*(IV)(OH)**$_2$, **5ME*SS*(IV)(OH)**$_2$ and **56ME*SS*(IV)(OH)**$_2$), and cisplatin at 24, 48 and 72 h. Data are expressed in RFU values. Data points signify mean ± SEM. n = 3 from three independent tests where samples were achieved in triplicate. Significance was identified by Tukey's multiple comparisons test for multiple comparisons. Group differences were considered statistically significant if * $p < 0.05$, ** $p < 0.01$, *** $p < 0.001$ and **** $p < 0.0001$ compared with cisplatin, as measured by one-way ANOVA.

HDAC Measurements

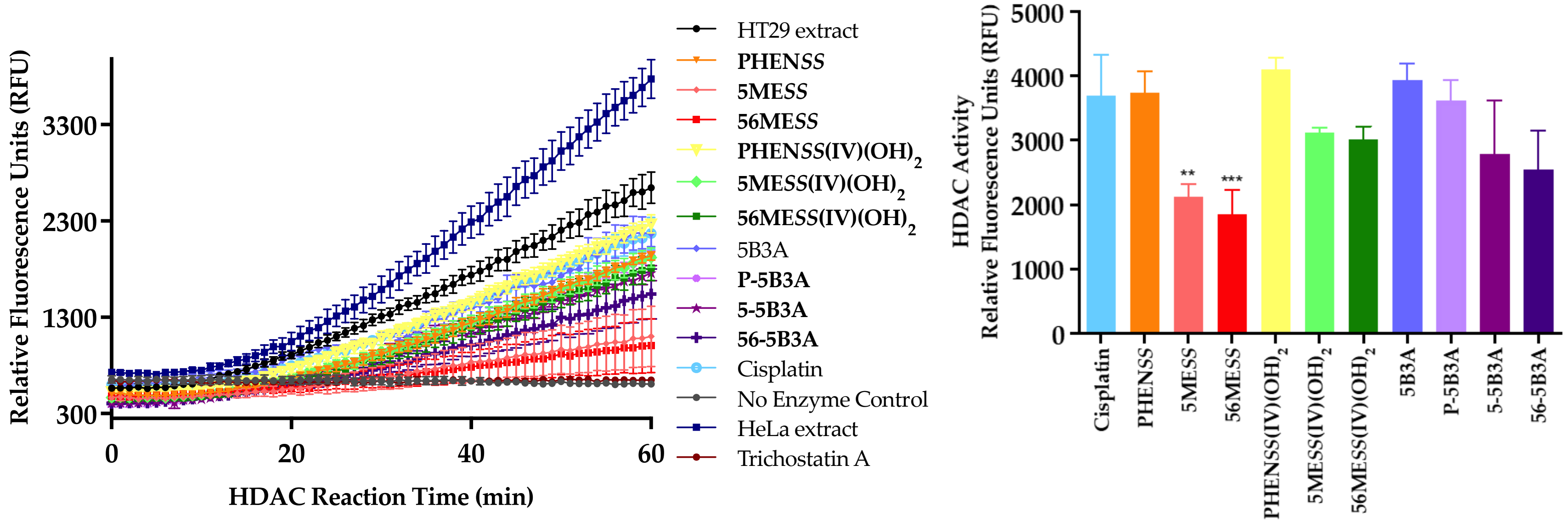

Figure S42: HDAC activity upon treatment with **P-5B3A**, **5-5B3A** and **56-5B3A**, 5B3A, the equivalent platinum(II) precursors (**PHEN*SS***, **5ME*SS*** and **56ME*SS***) and platinum(IV) scaffolds (**PHEN*SS*(IV)(OH)₂**, **5ME*SS*(IV)(OH)₂** and **56ME*SS*(IV)(OH)₂**), and cisplatin at 72 h. Data points signify mean ± SEM. n = 3 from three independent experiments where samples were conducted in triplicate. Significance was identified by Tukey's multiple comparisons test for multiple comparisons. Group differences were considered statistically significant if ** $p < 0.01$ and *** $p < 0.001$ compared with cisplatin, as measured by one-way ANOVA.
